# Positron emission tomography imaging of novel AAV capsids maps rapid brain accumulation

**DOI:** 10.1038/s41467-020-15818-4

**Published:** 2020-04-30

**Authors:** Jai Woong Seo, Elizabeth S. Ingham, Lisa Mahakian, Spencer Tumbale, Bo Wu, Sadaf Aghevlian, Shahin Shams, Mo Baikoghli, Poorva Jain, Xiaozhe Ding, Nick Goeden, Tatyana Dobreva, Nicholas C. Flytzanis, Michael Chavez, Kratika Singhal, Ryan Leib, Michelle L. James, David J. Segal, R. Holland Cheng, Eduardo A. Silva, Viviana Gradinaru, Katherine W. Ferrara

**Affiliations:** 1 Molecular Imaging Program at Stanford (MIPS), Department of Radiology, School of Medicine, Stanford University, Stanford, CA USA; 2 Department of Biomedical Engineering, University of California, Davis, CA USA; 3 Department of Molecular and Cellular Biology, University of California, Davis, CA USA; 4 Division of Biology and Biological Engineering, California Institute of Technology, Pasadena, CA USA; 5 Department of Bioengineering, Stanford University, Stanford, CA USA; 6 Stanford University Mass Spectrometry, Stanford, CA USA; 7 Genome Center and Department of Biochemistry and Molecular Medicine, University of California, Davis, CA USA

**Keywords:** Molecular imaging, Positron-emission tomography, Viral tracing

## Abstract

Adeno-associated viruses (AAVs) are typically single-stranded deoxyribonucleic acid (ssDNA) encapsulated within 25-nm protein capsids. Recently, tissue-specific AAV capsids (e.g. PHP.eB) have been shown to enhance brain delivery in rodents via the LY6A receptor on brain endothelial cells. Here, we create a non-invasive positron emission tomography (PET) methodology to track viruses. To provide the sensitivity required to track AAVs injected at picomolar levels, a unique multichelator construct labeled with a positron emitter (Cu-64, t_1/2_ = 12.7 h) is coupled to the viral capsid. We find that brain accumulation of the PHP.eB capsid 1) exceeds that reported in any previous PET study of brain uptake of targeted therapies and 2) is correlated with optical reporter gene transduction of the brain. The PHP.eB capsid brain endothelial receptor affinity is nearly 20-fold greater than that of AAV9. The results suggest that novel PET imaging techniques can be applied to inform and optimize capsid design.

## Abstract (web summary)

Adeno-associated viruses (AAVs) can be targeted in a tissue-specific manner, but their tissue accumulation cannot be assessed in a non-invasive manner. Here the authors conjugate a multivalent chelator labelled with Cu-64 to the surface of AAVs and image the brain accumulation of the PHB.eB capsid by PET.

## Introduction

Therapeutic delivery to the brain has traditionally been limited in volume. The background level of protein/nanotherapeutics reaching the brain is on the order of 0.1 percent injected dose per cubic centimeter (% ID/cc)^1^, necessitating more efficient methods of delivery. Engineered adeno-associated viruses (AAVs), single-stranded deoxyribonucleic acid (ssDNA) encapsulated within 25-nm protein capsids, have recently shown potential to greatly increase transduction as compared with previous therapeutics^2–4^. AAVs can infect dividing and non-dividing cells and result in highly efficient long-term transduction in a broad range of tissues^5,6^. This is particularly significant as AAV gene therapy has a solid safety profile, was first approved by the FDA in December 2017^7^ and more than 200 clinical trials have been conducted since 1989^8^. Recently, AAVs have been shown capable of delivering CRISPR-Cas9 gene silencing in vivo^9^, expanding their potential utility. Using a directed evolution approach to viral capsid engineering and selection, AAV-PHP.eB, containing a 2-mer substitution and 7-mer peptide insertion in a surface exposed loop of the capsid, enhanced neuronal transduction throughout the brain compared to the conventionally used AAV serotype 9 (AAV9)^3^. This 40 to 90-fold increased efficiency is believed to result from a novel interaction between virus and the brain endothelial cell receptor LY6A^10,11^.

In vivo imaging has great potential to contribute to the design and optimization of AAVs. The biodistribution of viral vectors has previously been evaluated by real-time PCR, Southern blotting of the transduced gene, western blotting, immunohistochemistry (IHC), and in vivo imaging of reporter proteins^12^. Many of these methods are invasive, relying on small quantities of tissue at a single site and/or time point^13^. In vivo imaging can determine the reporter protein level expressed from a transduced gene across an entire region of interest (ROI) over time; however, the underlying mechanisms for differences in the reporter protein cannot be directly identified with this approach. Development of a labeling method for non-invasive pharmacokinetics (PK) studies is valuable for several reasons. First, in vivo imaging can directly and non-invasively assess endothelial receptor binding at multiple time points. Second, PK can be non-invasively assessed even with repeated administration, the potential for which increases since capsid engineering and cargo development also address issues related to AAV neutralization and immunogenicity^5,14,15^. Third, quantitative imaging techniques facilitate comparisons across strains and species.

We therefore set out to develop an imaging method to track therapeutic viral constructs and quantify their binding to endothelial surface receptors. Positron emission tomography (PET) provides an ideal non-invasive method to track viral constructs in brain-related and other diseases^16^. In particular graphical analysis of plasma and tissue radiotracer uptake at multiple time points produces a linear plot, the slope of which is related to the number of available tracer binding sites. PET facilitates the interpretation of endothelial binding and the quantification of reversible receptor binding^17,18^. This provides a unique noninvasive assessment of AAV uptake.

PET imaging has not previously been applied for systemic AAV tracking. Surface modification of AAVs has previously focused on tagging fluorophores^19–24^ to PEG^25,26^, or adding peptides^27,28^, antibodies^23^, or small molecules^29^ to the capsid. Given that most earlier generations of AAV and other viral therapies were not designed for specific organ targeting, imaging studies labeled multiple lysines on the capsid with a lesser impact on organ-specific endothelial targeting and transduction^30^. Recently, the AAV capsid was labeled with I-124, but the study was limited to direct intracranial injection to the brain and therefore did not focus on the receptor binding characteristics or endothelial accumulation^31^. Alternatively, reporter gene imaging has been used to measure transduction but cannot quantify PK^32^. Thus, our study fills a void in PET imaging of the PK of novel capsids.

**Fig. 1:**
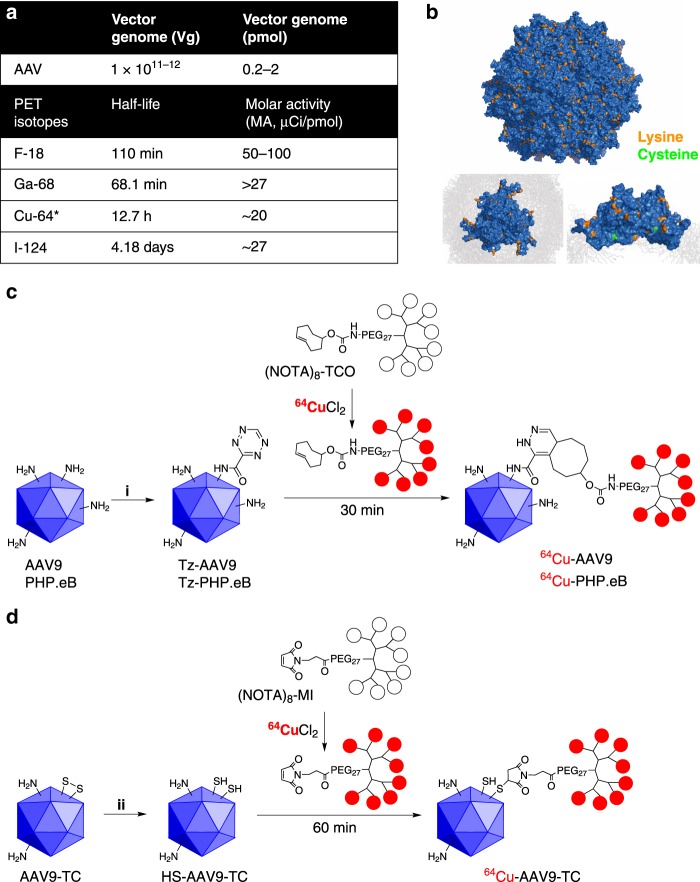
Strategy for labeling AAVs with a positron emitter. **a** Table presents the number of AAVs systemically injected and the molar activity of positron emitters. **b** Solvent accessible surface of AAV9 capsid^30^ (PDB ID:3UX1) displayed by PyMOL software. Insets highlight a trimer around a threefold axis. Orange and green color represent lysine and cysteine residues, respectively. Solvent radius is set as 1.4 angstrom. AAV capsid is composed of 60 structurally identical viral protein subunits (VPs) with 1:1:10 ratio of VP1:VP2:VP3. **c**, **d** are the labeling schemes of AAV-PHP.eB, AAV9, and AAV9-TC. **c** Surface modification with multichelators (MC) on lysine residues in capsids. (NOTA)_8_-TCO (incorporating a PEG_27_ spacer) is employed for the radiolabeling of Tz-AAV9 or Tz-PHP.eB after reaction of Tz-NHS ester with AAV9 or PHP.eB. (i, tetrazine-PEG_5_-NHS (Tz-NHS) ester, 1x PBS (pH 8), 4 °C, overnight dialysis in 20 kDa molecular weight cut-off (MWCO) membrane). **d** The site-specific radiolabeling on cysteine residues in AAV9-TC was employed with the multichelator-maleimide conjugate ((NOTA)_8_-MI) incorporating Cu-64 after the reduction of tetracysteine with TCEP (ii, TCEP in 1x PBS (pH 7.0–7.5)). Asterisk indicates average molar radioactivity of Cu-64 from commercial vendor as used in this study.

The challenge in monitoring the PK of systemically injected AAVs with PET (particularly with high time resolution) is to achieve a trackable level of radioactivity while matching the half-life of the positron emitter to AAV circulation half-life, which ranges from minutes to days^33^. An additional challenge is to minimize conjugation to key AAV surface features. High molar activity (MA) positron emitters, such as F-18 and Ga-68, typically have a short half-life (*t*_1/2_ of 110 and 68 min, respectively); thus, limiting their utility (Fig. 1a). The dose for systemic administration of AAVs in mice is low; ~10^11–12^ vector genomes (vg) are injected, corresponding to 0.2–2 pmol of AAVs. Cu-64 has a half-life of 12.7 h and is therefore well suited to the AAV half-life in blood^34^; however, combining ^64^CuCl_2_ (MA, ~20 μCi/pmol) and 2 pmol of AAVs yields ~40 μCi of labeled AAVs when the labeling ratio of Cu-64 to AAVs is 1:1. Real-time high resolution imaging is impaired with this very low level of radioactivity. In order to facilitate high signal-to-noise (SNR) PET imaging at a low AAV dose, we have therefore synthesized a bifunctional multichelator that increases the MA of ^64^Cu/molecule up to 10 times compared to a single chelator.

Our study highlights the potential to use PET imaging to track viral capsids after systemic injection, facilitating noninvasive quantitation of organ accumulation and clearance and endothelial receptor binding. The multichelator approach developed here is applied for optical microscopy, system-level PET imaging and autoradiography. Based on these analyses, we find that brain accumulation of PHP.eB, a novel AAV9 derivative with high brain tropism, exceeds that reported in previous PET studies of brain uptake of targeted therapies. Further, the high signal-to-noise ratio obtained with the multichelator approach can be exploited to quantify endothelial receptor affinity over the first 30 min after injection. Here, brain affinity of the PHP.eB capsid is enhanced nearly 20-fold as compared with the well-established AAV9 capsid. Most importantly, the labeling method retains the transduction efficacy of the AAV and can be applied in future studies to inform and optimize the design of AAVs and other viral capsids.

## Results

### Syntheses of multichelators

We have developed a bio-orthogonal approach for coupling a multichelator and AAV, based on conjugation to AAV surface lysines and cysteines and used this approach to compare the PK of AAV9-PHP.eB (AAV9-PHP.eB is denoted as PHP.eB hereafter) with AAV9 and AAV9-tetracysteine (AAV9-TC). Conjugation to surface lysines was previously shown to be feasible in fluorescence imaging where AAV surface lysines were modified with a fluorescent dye, which facilitated AAV tracking without hampering transduction efficiency^19–22,35^. Based on surface solvent accessibility in the X-ray structure of the AAV9 capsid^30^, the estimated number of exposed lysines on AAV9 and PHP.eB ranges from 420 to 480 out of 1185 and 1245 total lysines, respectively (Fig. 1b). This includes 7–8 lysines per viral protein (VP), with one viral particle composed of 60 units of VP. We based the surface lysine labeling strategies on inverse electron demand Diels–Alder reactions (IEDDA), which offer a fast, quantitative (>50,000 M^−1^S^−1^) orthogonal reaction^36^. We modified a small number of the surface lysines with Tz-NHS ester, followed by conjugation of the ^64^Cu-multichelator-transcyclooctene (TCO), (NOTA)_8_-TCO (Fig. 1c). Multichelator-maleimide, (NOTA)_8_-MI, was employed (Fig. 1d) to label AAV9-TC. Notation describing the labeled AAVs (e.g. ^64^Cu-PHP.eB) is defined in Fig. 1c, d. In order to confirm that the lysine attachment does not alter receptor binding, we compared labeling with this approach to labeling of cysteines available on a tetracysteine (TC) engineered version of AAV9 using a maleimide-thiol reaction of the multichelator (Supplementary Fig. 1). In this virus, an (HRWCCPGCCKTF) motif was inserted at amino acid 139 (residue numbered by VP1 sequence) of the AAV9 capsid proteins (AAV9-139). The insertion site is located in the N-terminal disordered region of capsid proteins VP1 and VP2. A similar modification was made in AAV9 and was shown to be accessible by labeling reagents without compromising viral integrity^24^.

The multichelators, (NOTA)_8_-TCO and (NOTA)_8_-MI, were synthesized through a solid phase reaction. Multistep coupling of Fmoc-Lys(Fmoc)-OH from polyethylene glycol(PEG)_27_-Lys(Boc) on resin afforded eight branched amines, further coupled with *tert-*Bu-NOTA-OH. A PEG spacer was included to separate the multichelator and reactive functional group. After cleavage of (NOTA)_8_-NH_2_ (Supplementary Fig. 2a, 1) from the resin, 1 was further functionalized to (NOTA)_8_-TCO (Supplementary Fig. 2a, 2) and (NOTA)_8_-MI (Supplementary Fig. 2a, 3). In all, 2 and 3 were isolated by HPLC presented monoisotopic mass peaks at 5026.67 (calculated mass: 5026.85 Da) and 4937.66 (calculated mass: 4937.74 Da) in MALDI mass analysis (Supplementary Fig. 2a), respectively. For optical studies of PHP.eB and AAV9 conjugated with the multichelator, (NOTA)_8_-A555-TCO with a cysteine introduced to conjugate A555-maleimide was synthesized as shown in Supplementary Fig. 2b. The mass (M + H^+^) of (NOTA)_8_-cys(SH)-NH_2_ (4), (NOTA)_8_-A555-NH_2_ (5) and (NOTA)_8_-A555-TCO (6) verified with MALDI mass analysis were detected at 4874.54 (calculated mass: 4875.70 Da), 5844.85 (calculated mass: 5843.94) and 6245.47 (calculated mass: 6243.16), respectively.

### Efficiency of Cu-64 incorporation on single and multichelator

To assess the incorporation of copper on the multichelator, increasing amounts of (NOTA)_8_-TCO were reacted with a known amount of nonradioactive Cu-63 spiked with the radioactive Cu-64, and the incorporation ratio was then compared with that achieved with the single chelator (NOTA-TCO). We confirmed that more than two single chelators, NOTA-TCOs, are required to incorporate one copper molecule (Supplementary Fig. 3a), and one multichelator, (NOTA)_8_-TCO, incorporated 5–8 copper molecules (Supplementary Fig. 3b). Thus, the multichelator achieves ~10 times higher molar radioactivity than the single chelator (Supplementary Fig. 3).

### Capsid surface modification maintains transduction efficiency

**Fig. 2:**
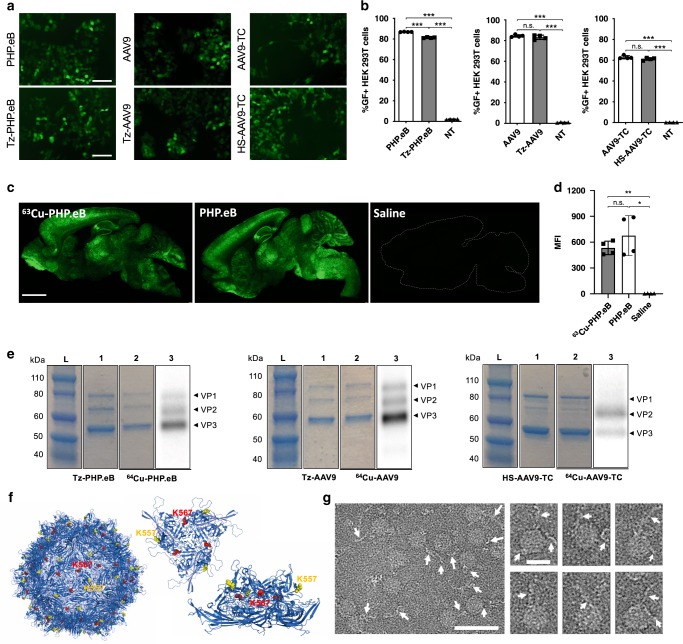
Transduction and labeling efficiency of surface modified AAVs. **a** Fluorescence microscopy images of human embryonic kidney(HEK) 293T cells after 24 h incubation with intact AAVs (upper row, PHP.eB, AAV9, and AAV9-TC) and corresponding modified AAVs (lower row, Tz-PHP.eB, Tz-AAV9, and HS-AAV9-TC) at 1 × 10^6^ AAV/cell. **b** Percentage of green fluorescent positive (GF^+^) HEK293T cells 2 days after incubation with unmodified AAVs (PHP.eB, AAV9, and AAV9-TC, white bar with black circles) and the corresponding modified AAVs (gray bar with black squares), assessed by flow cytometry. The frequency of GF^+^ cells treated with unmodified and modified AAVs was similar and distinct from non-treated (NT, black triangles) cells (*n* = 4 per group). **c** Representative GFP images of sagittal brain sections from a C57BL/6 mouse at 3 weeks after tail vein administration of ^63^Cu-PHP.eB, PHP.eB (1.5 × 10^10^ vg) or saline (negative control) and **d** mean fluorescence intensity (MFI) of sagittal brain sections (^63^Cu-PHP.eB: gray bar with black squares, PHP.eB: white bar with black circles, saline: black triangles, *n* = 4). **e** SDS-PAGE of modified AAVs (Tz-PHP.eB, Tz-AAV9, and HS-AAV9-TC; lane 1) and radiolabeled AAVs (^64^Cu-PHP.eB, ^64^Cu-AAV9, and ^64^Cu-AAV9-TC; lane 2 and 3). The three bands depict viral protein (VP) 1–3 (L: standard protein ladder). Lane 1–3 illustrate blue-stained VPs (lanes 1 and 2) and radiolabeled VPs (lane 3), respectively. **f** Illustration of AAV9 capsid with modified lysines. Left: full view of AAV9, middle and right: top and side views of trimer viral proteins, respectively. The K557 (yellow) and K567 (red) lysine residues are highlighted. **g** Field view of direct-electron cryoEM images of PEG(40 kDa)-AAV9 (left image) and enhanced projection images of selected PEG(40 kDa)-AAV9 capsids (six right images). White arrows mark the 40 kDa PEG molecules extended from the selected AAV capsids. Data are shown as mean ± SD. Brown-Forsythe and Welch ANOVA with Dunnett’s T3 multiple comparison test compares means (**b**, **d**). Significance is presented as n.s. (not significant), *P ≤ 0.05, **P ≤ 0.01, and ***P ≤ 0.001. Whole gel and gel autoradiography images and *P* values are shown in the source data. Scale bars: 100 μm (**a**), 2 mm (**c**), 50 nm (**g**, left), 20 nm (**g**, right).

To determine the maximum molar ratio of Tz-NHS and (NOTA)_8_-TCO that can be incubated with PHP.eB:*CAG-GFP* (and similarly (NOTA)_8_-MI with AAV9-TC:*CAG*-*mNeonGreen*) without hampering its integrity, we monitored the AAV transduction efficiency in HEK293T cells before and after labeling (Fig. 2a, b, Supplementary Figs. 4 and 5). In previous studies, the conjugation of NHS (succinimidyl ester) to AAVs typically proceeded under strong basic conditions (0.1 M Na_2_CO_3_, pH 9.3); however, the reported procedures have been inconsistently detailed, and the experimental conditions vary widely (summarized in Supplementary Table 1). To avoid harsh conditions, the reaction was performed at pH 8 by mixing PBS and Na_2_CO_3_ (v:v, 8:2), as is routinely exploited in preparation of antibody conjugates. Under this reaction condition, PHP.eB (4.2 × 10^11^ vg, 0.7 pmol particles) was incubated with Tz-NHS and followed an IEDDA reaction with (NOTA)_8_-TCO (Supplementary Fig. 4a). SDS-PAGE analysis of PHP.eBs labeled with (NOTA)_8_-TCO clearly showed three VP bands with similar molecular weight to the unmodified control PHP.eB (Supplementary Fig. 4b). Incubation with a molar ratio of 500 and above resulted in additional high molecular-weight protein bands (Supplementary Fig. 4b) and a significant reduction in fluorescent-protein expressing cells (Supplementary Fig. 4c). Both assays suggest that keeping the molar ratio of (NOTA)_8_-TCO/PHP.eB below 500-fold maintains transduction efficiency of HEK293T cells and prevents the aggregation of capsid proteins after labeling of PHP.eB. Limits on the concentration of the chelator were more restrictive with AAV9-TC. AAV9-TC:*CAG-mNeonGreen* (5.8 × 10^12^ vg, 9.6 pmol) after reduction to HS-AAV9-TC by TCEP was reacted with (NOTA)_8_-MIs at 14, 70, and 140 pmol (Supplementary Fig. 5a). Multiple bands of over-labeled VPs were found when the incubated (NOTA)_8_-MI/AAV9-TC ratio was 70-fold or more (Supplementary Fig. 5b). For AAV9-TC, multiple bands likely result from the non-specific maleimide conjugation with primary amines as previously reported^37^. In this previous report, a similar protein band shift occurred in SDS-PAGE at dye:protein ratios >40:1. Irrespective of the multiple band formation, AAV9-TC transduction efficiency was preserved at all levels of modification (Supplementary Fig. 5c).

Under the optimized conditions, AAV9 and PHP.eB were then labeled with multichelators that have been conjugated with Cu-64 following the procedure detailed in the Methods section. Transduction efficiencies of Tz-AAV9 and -PHP.eB (modified from AAV9:*CAG-mNeonGreen* and PHP.eB:*CAG-GFP* with Tz-NHS, respectively) and HS-AAV9-TC (a reduced form of AAV9-TC:*CAG-mNeonGreen*) were then compared with those of intact AAVs in HEK293T cells in vitro (1 × 10^6^ vg/cell). As assessed by fluorescent green protein expression in HEK293T cell images, fluorescent-protein transduction was similar at 24 h after incubation with intermediates before and after modification (Fig. 2a). Flow cytometry at 48 h confirmed the comparable transduction efficiency of modified and unmodified AAVs for Tz-AAV9 and -PHP.eB and HS-AAV9-TC (Fig. 2b). Most importantly, the transduction efficiency of ^63^Cu-labeled PHP.eB:*CAG-GFP* and PHP.eB:*CAG-GFP* in C57BL/6 mice was evaluated to determine whether the multichelator influenced viral delivery and GFP production in the brain at 3 weeks after tail vein administration (1.5 × 10^10^ vg). The GFP mean fluorescence intensity (MFI) within the brain was similar (Fig. 2c, d, *n* = 4, n.s.) following injection of the unmodified (PHP.eB) or the labeled AAV (^63^Cu-PHP.eB) and undetectable after saline injection. Taken together, the results demonstrate that our optimized labeling condition preserved the AAV’s functional properties.

### Characterization of radiolabeled capsid on viral proteins

The viral protein (VP) bands were visualized via protein staining (blue, 1st and 2nd lane), and the radiolabeled VP bands were imaged with sodium dodecyl sulfate-polyacrylamide gel electrophoresis (SDS-PAGE) and autoradiography (gray, 3rd lane) (Fig. 2e). The band location of the three VPs (blue bands) between Tz-PHP.eB and ^64^Cu-PHP.eB, Tz-AAV9 and ^64^Cu-AAV9 and HS-AAV9-TC and ^64^Cu-AAV9-TC were similar. The relative radiolabeling of VP3 was greater than VP1 or VP2 for PHP.eB and AAV9 (Fig. 2e), directly related to the ratio of protein abundance for the three VPs, 1:1:10 (VP1, VP2, VP3). AAV9-TC was generated by site-specific insertion of the HRWCCPGCCKTF peptide motif at the VP1/VP2 interface at the 139^th^ amino acid^24^. As a result, the gel image from autoradiography of ^64^Cu-AAV9-TC showed the VP2 band as the major radiolabeled VP whereas the protein staining (blue) of ^64^Cu-AAV9-TC was similar to the ratio of each VP (VP1:VP2:VP3, 1:1:10) (right column image of Fig. 2e).

### PHP.eB size was unchanged after ^63^Cu-multichelator labeling

The size of ^63^Cu-labeled and unlabeled PHP.eB was 27.9 ± 0.64 and 27.1 ± 0.68 nm (*n* = 3), respectively, and detected as a single peak (Supplementary Fig. 6).

### Proteomic analysis of modified lysines on the capsid protein

We first determined the lysine sites modified with Tz-NHS by proteomic analysis. Mass lists from the analyses of excised gel bands of VP1, VP2, and VP3 after reaction of PHP.eB with Tz-NHS showed that tetrazines were predominantly incorporated on two lysines (K557 and K567) (Supplementary Table 2), which exist in all VPs. Importantly, the lysine at the 595th amino acid located within the engineered peptide sequences (DGTLAVPFK), critical for the distribution of PHP.eB to the brain and transduction of its transgene^3^, remained unreacted. Based on the crystal structure of AAV9^30^, neither of the modified sites are located in the core of the threefold-proximal spikes, the region considered to be responsible for most virus-host interactions. K567 is located in the valley between the spikes, while K557 is on the distal shoulder (Fig. 2f). To the best of our knowledge, the two sites have not been reported to be involved in receptor binding for AAV9 derivatives.

### Determination of the number of AAV labels

We compared results from optical labeling and electron microscopy to determine the number of labels. We examined the number of labels per capsid using the multi-armed fluorescent label (Supplementary Figs. 2b and 7a) combined with quantification of the AAV concentration through a titer. We applied this approach for both the amine- and thiol-directed coupling approaches. Labeling with 200–350 equivalents of Tz-NHS and reaction with 10–15 equivalents of (NOTA)_8_-A555-TCO gave 5.4 ± 2.3 (*n* = 6) and 3.5 ± 3.0 (*n* = 8) labels per PHP.eB and AAV9 capsid, respectively (Supplementary Table 3). AAV9-TC reduced by 100 equivalents of TCEP and subsequently reacted with 20 equivalents of A555-C2 maleimide yielded 0.5 ± 0.3 (*n* = 4) ea/vg of A555-AAV9-TC (Supplementary Table 3).

Furthermore, since the 5 kDa size of the (NOTA)_8_-A555 conjugate on AAV9 was not reliably visualized on cryogenic electron microscopy (cryoEM), we conjugated a larger label (PEG(40 kDa)-TCO) (Supplementary Fig. 7b) to the capsid. This label was conjugated to AAV9 (denoted PEG(40 kDa)-AAV9) using the same conditions used for the in vivo imaging and was used to visualize the number of labels per virus (Supplementary Fig. 7a). PEG(40 kDa)-AAV9 was obtained from a reaction with Tz-AAV9 and 4 equivalents of PEG(40 kDa)-TCO and showed one to three labels per capsid on cryoEM images (Fig. 2g).

### AAV radiolabeling was achieved with high radiochemical purity

The 20–35 pmol of (NOTA)_8_-TCO and (NOTA)_8_-MI were enough to incorporate >99% of 1–2 mCi Cu-64. In situ incubation of Tz-AAV9 and -PHP.eB and HS-AAV9-TC with these multichelators yielded ^64^Cu-AAV9, -AAV9-TC, and -PHP.eB to 2.2% (1.78 MBq (48 μCi)), 6.6% (4.88 MBq (132 μCi) and 7.5 ± 6% (3.9 ± 1.7 MBq (106 ± 45 μCi)), respectively (decay corrected). Radiochemical purity of radiolabeled AAVs on instant radio-thin layer chromatography was above 98%.

### PET imaging quantified brain accumulation and receptor binding

**Fig. 3:**
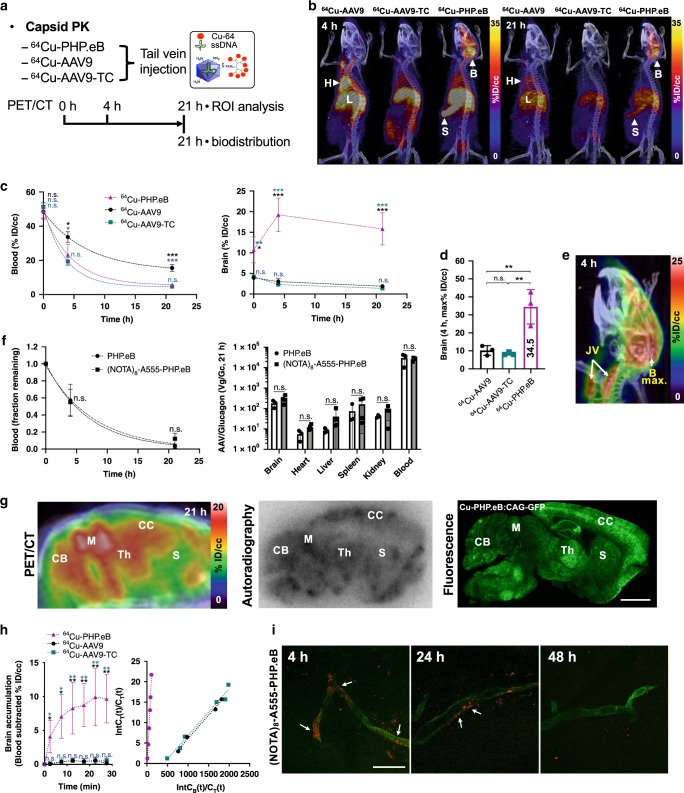
PET and optical imaging-based assessment of AAV pharmacokinetics in C57BL/6 mice. **a** Experimental setup for region of interest (ROI) analysis (0, 4, and 21 h) and biodistribution (21 h) of ^64^Cu-PHP.eB, -AAV9, and -AAV9-TC. PET images are acquired at 0, 4 and 21 h after AAV tail vein administration. **b** Projected PET/CT images at 4 (left) and 21 h (right) (H heart, L liver, S spleen, B brain). **c** Time activity curves (over 21 h) and **d** maximum brain uptake (at 4 h) of ^64^Cu-PHP.eB (magenta triangle), ^64^Cu-AAV9 (black circle), and ^64^Cu-AAV9-TC (turquoise square) from the ROI analysis of blood and brain (*n* = 3) after tail vein administration. **e** Representative projected PET/CT image at 4 h of ^64^Cu-PHP.eB within the brain (B brain, JV jugular vein). **f** PK (left) and 21-h biodistribution (right) of PHP.eB (*n* = 3, black circle) and (NOTA)_8_-A555-PHP.eB (*n* = 4, black squares) obtained by qPCR. **g** Sliced PET/CT, autoradiography and GFP images of sagittal section of mouse brain (CB cerebellum, M midbrain, Th thalamus, CC cerebral cortex, S striatum) acquired at 21 h, 21 h and 3 weeks, respectively, after tail vein injection of ^64^Cu-PHP.eB for PET/CT and autoradiography and non-radioactive ^63^Cu-PHP.eB for the GFP image. **h** ^64^Cu-AAVs brain accumulation (*n* = 3 per group) measured 30 min after tail vein administration (left) and Logan plots (right) of brain uptake rate after AAV administration. **i** Representative confocal images of (NOTA)_8_-A555-PHP.eB (red) on brain endothelium (green) acquired 4, 24, and 48 h after tail vein injection. White arrows indicate (NOTA)_8_-A555-PHP.eBs (red). Data are shown as mean ± SD. One-way ANOVA with Tukey’s multiple comparison test (**c**, **d**, and **h** (left)) compared means of the three groups. Multiple unpaired *t-*tests with the Holm-Sidak method with alpha = 0.05 compared the means in **f**. Significance: n.s. (not significant), *P ≤ 0.05, **P ≤ 0.01, and ***P ≤ 0.001. *P* values are shown in the source data. Intensity values in **b**, **d**, **e**, and (**g**, left) are percent injected dose per cubic centimeter (% ID/cc). Scale bars: 2 mm (**g**), 25 μm (**i**).

The PK and biodistribution of the ^64^Cu-AAV9, -AAV9-TC, and -PHP.eB capsids (as defined in Fig. 1) were assessed in C57BL/6 mice (*n* = 3/group) with PET/CT as illustrated in Fig. 3a. The projection images acquired of AAVs revealed two remarkable distinctions: the high brain uptake of PHP.eB and the extended blood circulation of AAV9 (Fig. 3b, Supplementary Movies 1–3). Blood circulation of AAV9 (*t*_*1/2*_ = 5.0 h) was longer than that of AAV9-TC (*t*_*1/2*_ = 2.4 h) and PHP.eB (*t*_*1/2*_ = 3.1 h) (Fig. 3c, Supplementary Tables 4 and 5). The faster clearance of PHP.eB from blood, as compared to AAV9, is expected due to rapid uptake within the brain (Supplementary Movie 2a at 4 h). The mechanism for the enhanced clearance of AAV9-TC has not been fully characterized; however, the tetracysteine motif (HRWCCPGCCKTF) on AAV9-TC can remain reactive after reduction by TCEP, and S-thiolation by serum proteins^38^ can reduce stability, potentially resulting in a protein corona or aggregation over time^39^. Thus, while the initial (30 min) receptor binding is expected to be similar, clearance from blood through the liver and intestine (over hours) is expected to be enhanced for AAV9-TC (Supplementary Movie 3a at 4 h). Brain accumulation of PHP.eB was greater at all time points than that of AAV9 (*n* = 3, *P* = 0.0096 at 0 h, *P* = 0.0004 at 4 h, *P* = 0.0007 at 21 h) and AAV9-TC (*n* = 3, *P* = 0.0116 at 0 h, *P* = 0.0003 at 4 h, *P* = 0.0006 at 21 h) (Fig. 3c, Supplementary Table 6). Maximum uptake (% ID/cc) of ^64^Cu-PHP.eB in the entire brain was 35% ID/cc, with the spatial maximum observed in the midbrain (Fig. 3d) and strong midbrain uptake clearly visualized in the projected PET/CT brain image (Fig. 3e). In addition, in order to assess the PK of multichelator-labeled PHP.eB compared to that of PHP.eB, we performed classical qPCR. Un-labeled PHP.eB and (NOTA)_8_-A555-PHP.eB cleared at a similar rate from the blood pool over 21 h (*t*_1/2_ = 4.8 h vs 5.3 h, respectively) (Fig. 3f, left, Supplementary Tables 4 and 7) and the blood clearance was similar to that observed by PET (Fig. 3c). The biodistribution of PHP.eB and (NOTA)_8_-A555-PHP.eB was similar in the major organs such as the brain, heart, liver, spleen, kidney and blood (Fig. 3f, right, Supplementary Table 8).

Sliced sagittal brain images in the cerebral cortex, thalamus, midbrain and cerebellum from PET/CT (after 21 h), autoradiography (after 21 h), and ex vivo GFP fluorescence (after 3 weeks) showed a consistent distribution of the viral capsid tag and corresponding transduced GFP protein expression (Fig. 3g, Supplementary Fig. 8). PHP.eB accumulation in the brain was then analyzed in dynamic 5-min intervals over the first 30 min. 4% and 10% ID/cc of PHP.eB was bound at 5 and 30 min, respectively, whereas AAV9 and AAV9-TC accumulation was <1% ID/cc (Fig. 3h, Supplementary Fig. 8). Furthermore, Logan plots, based on a reversible accumulation model, demonstrated that the 30 min accumulation in brain was greater for PHP.eB»AAV9 ~ AAV9-TC, with a distribution volume of 0.210, 0.011, and 0.011, respectively (Fig. 3h). Thus, the affinity of PHP.eB for the brain endothelium is estimated to be 20-fold higher than for AAV9 and AAV9-TC. The receptor affinity of AAV9 was identical for the two labeling methods as assessed by the initial 30-min Logan plot (Fig. 3h).

### Multichelator does not alter PHP.eB endothelial accumulation

We employed two optical probes: a probe in which an optical dye (A555-NHS ester) was attached to the native capsid lysines and a second optical probe ((NOTA)_8_-A555-TCO) conjugated to the multichelator construct in a manner similar to the (NOTA)_8_-TCO conjugate (Supplementary Fig. 7a). The binding of A555-PHP.eB, A555-AAV9 (Supplementary Fig. 9), (NOTA)_8_-A555-PHP.eB (Fig. 3i) or (NOTA)_8_-A555-AAV9 (Supplementary Fig. 10) to the brain endothelium, observed by confocal microscopy at 4, 24, and 48 h after injection (Z-stack images in Supplementary Movie 4), showed that punctate clusters were observed at 4 h after injection. The fluorescence intensity gradually diminished by 24 h and was similar to saline injection (Supplementary Fig. 10) at 48 h. Taken together, the optical and PET images suggest that the early-bound PHP.eB crossed the BBB within 48 h and specific and effective binding of PHP.eB to the brain endothelium was confirmed.

### PET imaging elucidates strain and treatment-dependent PK

**Fig. 4:**
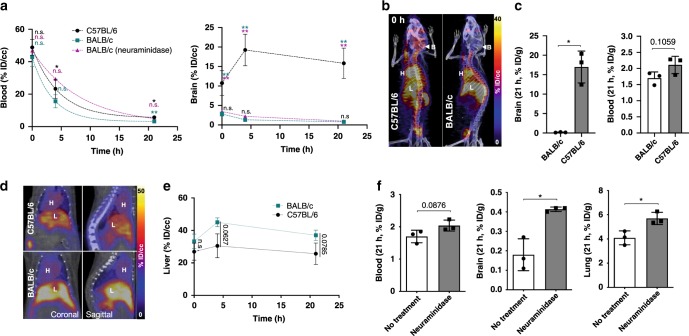
Strain and neuraminidase-dependent pharmacokinetics of ^64^Cu-PHP.eB. **a** Time activity curves of PHP.eB obtained from region of interest (ROI) analysis of blood (left) and brain (right) from C57BL/6 (black circle), BALB/c (turquoise square), and neuraminidase-treated BALB/c (magenta triangle) mice over 21 h (*n* = 3 per group). Radioactivity from ROI analysis is presented as % ID/cc. **b** Representative PET/CT projection images (B brain, H heart, L liver) acquired over 30 min after tail vein injection of ^64^Cu-PHP.eB to C57BL/6 and BALB/c mice. **c** Biodistribution (% ID/g) of ^64^Cu-PHP.eB in brain (left) and blood (right) in C57BL/6 (gray bar with black squares) and BALB/c (white bar with black circle) mice at 21 h (*n* = 3). **d** Sliced PET/CT image (H heart, L liver) at 4 h after tail vein injection of ^64^Cu-PHP.eB. **e** Time activity curve of ^64^Cu-PHP.eB measured from C57BL/6 (black circle) and BALB/c (turquoise square) livers (*n* = 3). **f** Biodistribution (21 h) of ^64^Cu-PHP.eB in blood (left), brain (middle), and lung (right) from BALB/c with no treatment white bar with black circle) versus BALB/c mice treated with neuraminidase (gray bar with black square) (*n* = 3). Data are shown as mean ± SD. For statistical analysis, a one-way ANOVA with Tukey’s multiple comparison test in **a** was performed to compare means of three groups (C57BL/6 vs BALB/c: turquoise, C57BL/6 vs BALB/c (neuraminidase): magenta, BALB/c vs BALB/c (neuraminidase): black) at each time point. Unpaired two-tailed Welch’s *t*-test was performed in **c**, (**e**, 0, 4, and 21 h) and **f**. Significance is presented as *P ≤ 0.05, **P ≤ 0.01. *P* values are shown in the source data. Maximum and minimum intensity values of PET/CT images in **b**, **d** are presented as percent injected dose per cubic centimeter (% ID/cc).

Since mouse strain dependence of PHP.eB BBB transcytosis has been reported^10,40^, the PK, brain uptake and biodistribution of ^64^Cu-PHP.eB were assessed by comparing BALB/c and C57BL/6 mice (Fig. 4a, Supplementary Table 9). Dramatically-reduced brain uptake of ^64^Cu-PHP.eB was confirmed in BALB/c with respect to C57BL/6 mice from the time-activity curve over 21 h (Fig. 4a) (*n* = 3, *P* = 0.0048 at 0 h, *P* = 0.0002 at 4 h, *P* = 0.004 at 21 h) and PET/CT images at 0 h (Fig. 4b). Similar results (% ID/g) were observed in the brain radioactivity from the biodistribution at 21 h (Fig. 4c, *n* = 3, *P* = 0.0193). In addition, the uptake of PHP.eB was greater in the liver of BALB/c than C57BL/6 mice (*n* = 3, *P* = 0.0627 at 4 h) (Fig. 4d, e and Supplementary Table 10). While the enhanced liver accumulation is anticipated given the lack of brain accumulation, a strain-specific immune response has also been reported to enhance liver accumulation in the BALB/c strain^41,42^. Further, the circulation time of PHP.eB (3.1 h) in the BALB/c mouse (2.4 h) was slightly lower than that in C57BL/6 mice (Fig. 4a, Supplementary Table 4). The results reaffirm the reduced brain uptake in BALB/c mice, which, unlike C57BL/6 mice, lack the LY6A receptor that the engineered PHP.eB binds to^10^; however, other differences also exist in the PK between strains.

Previously, pretreatment with neuraminidase (NA) in vitro and in vivo, was reported to expose terminal N-linked galactose and enhance AAV9 binding^43,44^. In our study, nasal administration of NA was followed by IV injection of ^64^Cu-PHP.eB and biodistribution at 21 h in BALB/c mice. Lung and brain accumulation were increased by NA administration 1.4 (*n* = 3, *P* = 0.0229) and 2.0-fold (*n* = 3, *P* = 0.0364), respectively (Fig. 4f). The treatment by NA also increased the PHP.eB circulation time from 2.4 h to 5.6 h (Fig. 4a). However, the increased accumulation was relatively small as compared to the differences in brain accumulation of PHP.eB between strains.

## Discussion

We found that radiolabeling AAVs with a unique multichelator construct allows for a detailed and quantitative study of AAV biodistribution and pharmacokinetics. Following conjugation of a dendrimer-like radioactive tag to novel AAVs, the fraction of radioactivity delivered to the brain was extraordinarily high, thus demonstrating the potential use of AAVs as carriers. PET, with appropriate analytic methods, was applied to noninvasively quantify the endothelial receptor binding with a resolution of minutes. We found that Logan analysis is particularly important for characterizing the binding of novel AAV capsids as a rapid and largely irreversible accumulation occurs on the endothelium of the target tissue. No alternative technique can quantify binding of viral capsids in real time; such information is critically important for capsid engineering and for the identification of the endothelial receptors responsible for AAV accumulation and transcytosis.

Recent reports indicate that enhanced brain transduction by PHP.eB in C57BL/6 mice requires an LY6A receptor-mediated pathway that is independent of galactose and is absent in BALB/c mice and non-human primates^10^. ^64^Cu-PHP.eB can be exploited as a non-invasive tool to measure endothelium binding mediated by the LY6A receptor in various mouse strains and other species. Furthermore, the binding of serotype AAVs to the AAV receptor (AAVR), identified as a critical host factor for infection of naturally-occurring AAVs^45^, can be assessed by a simple blocking study with novel AAVs.

Optical imaging of the tagged capsid validated the accumulation of the capsids on the brain endothelium within minutes and the elimination of the punctate fluorescence on the endothelium over 24 h. Given the very small size of the AAVs (25 nm), tracking of their fluorescence within the brain was not feasible. PET imaging complemented the information in the optical image by demonstrating that the accumulated radioactivity remains within the brain over 21 h. Classical pharmacokinetic analysis based on a PCR further validated the brain accumulation. Given that the radioactivity was tagged to the capsid surface, the distribution of radioactivity throughout the brain suggests that the capsids were transcytosed across the BBB. This observation supports previous reports of AAV transcytosis across the BBB, as observed in vitro^46^. Combined PET and optical reporter gene imaging demonstrated that the pattern of transduction within the brain was similar to the distribution of the radioactive tag.

The stoichiometry of fluorophore-TCO conjugates on the surface of AAV9 and PHP.eB was on average four labels per particle. Labeling of a fluorescence-maleimide conjugate on AAV9-TC conferred 0.6 label per capsid. CryoEM of AAV9 conjugated with PEG(40 kDa) similarly showed 2–3 copies of extended PEG-string density per capsid. Our AAV surface modification with tetrazine-NHS ester retained transduction efficiency in HEK cells and in in vivo transduction studies. Following conjugation with the 5 kDa multichelator at a multichelator:AAV ratio of 1:2 PHP.eB, binding to the brain endothelium and transduction were maintained. Future work will focus on optimizing the number and size of the conjugated tags. Here, we minimized the number of tags per AAV in the in vivo studies of transduction to minimize any effect on transport. Given the relatively small loading capacity of AAVs, the potential to conjugate additional cargo to the capsid could be transformative. Multiple gene editing components, complementary therapeutics or additional imaging tags can be attached to the surface.

The predominantly-labeled lysines of each viral protein within PHP.eB were K557 and K567. K61, K92, K528, K618, K696, and K700 were modified in a smaller fraction of capsids (Supplementary Table 2). We speculate that K557 and K567 are susceptible to reaction with the NHS-ester and that this is the basis of their enhanced modifications. The variable region VII (aa545-aa558), including K557, is located within a region of the AAV capsid associated with liver transduction^47^ and delayed blood clearance^30,33^; however, to our best knowledge, there is no report on the direct involvement of K557 or K567 in host receptor binding. Recently, unnatural amino acids (UAA) bearing an azide were site-specifically engineered in AAV2 (at aa 587) and AAV-DJ (a derivative of AAV8, at aa 589) capsids and utilized to conjugate oligonucleotides^48^. Specific protocols can also be developed for labeling of other capsids. For example, there is a lysine adjacent to K557 on AAV2 and adjacent to K567 on AAV1, 6, 8, 9, and 10 (Supplementary Table 2). Therefore, addition or substitution of lysines, cysteines or UAAs within these sequences or other AAVs can also provide a unique labeling site.

Finally, the PET method developed here to monitor binding and pharmacokinetics will be paired with PET reporter gene imaging in future work. A PET reporter gene based on pyruvate kinase (PKM2) has been shown to have low background in the brain and can be packaged within AAVs^49^. PKM2 can be used with the reporter probe [^18^F]DASA-23^49^, which is permeable to the blood-brain barrier in order to monitor brain transduction over months or years. In the future, we will couple this tracer with the PET tag described here.

## Methods

### Materials and reagents

All solvents were purchased from Fisher Chemical, Sigma-Aldrich and Acros. The reagents and materials for multichelator synthesis were purchased from Novabiochem, Click Chemistry Tools and Biotage. PEG_27_ spacers were purchased from Chem-Impex International Inc. and Polypure. PEG(40 kDa)-amine (Creative PEGWorks) was purchased from Fisher Scientific. For the capsid SDS-PAGE, the gels, buffer, standard ladder and protein staining reagents were purchased from ThermoFisher Scientific. AFDye555-maleimide (Fluoroprobes), AlexaFluor555-NHS ester (ThermoFisher Scientific) and AlexaFluor555-C2-maleimide (ThermoFisher Scientific), each with 555 excitation max and 580 emission max, are denoted as A555 throughout. The detailed list of materials and reagents is in the Supplementary Methods.

### Cell line and AAVs

Human embryonic kidney cells (HEK293T) were obtained from ATCC (CRL-1573). AAV9, AAV9-TC, and PHP.eB packaging including *CAG-mNeonGreen* or *CAG-DIO-GFP* were prepared as described in the Supplementary Methods section entitled “Preparation of AAV9, AAV9-TC, and PHP.eB”. All AAVs were used within two months of preparation. AAV-PHP.eB packaging *CAG-GFP* was purchased from Addgene (#37825-PHP.eB). All AAVs used for in vitro/in vivo transduction, PET/CT, and optical studies are summarized in Supplementary Table 11.

### Synthesis of (NOTA)_8_-NH_2_

Branched (NH_2_)_8_-NH_2_ was synthesized on rink amide resins (0.49 mmol/g, 0.15 g) in a microwave-assisted solid phase synthesizer (Initiator + Alstra, Biotage) as shown in Supplementary Fig. 2a. Sequential coupling reaction was programed to be performed at 75 ^o^C for 5 min with Fmoc-lys(Boc)-OH (3 equivalents, 1.47 mmol, 113 mg), 0.2 M Fmoc-PEG_27_-OH (3 equivalents, 1.47 mmol, 372 mg), 0.2 M Fmoc-lys(Fmoc)-OH (3 equiv., 1.47 mmol, 131 mg), 0.2 M Fmoc-lys(Fmoc)-OH (5 equivalents, 2.45 mmol, 219 mg), and 0.2 M Fmoc-lys(Fmoc)-OH (4.9 equivalents, 439 mg) with 0.1 or 0.5 M HBTU (one equivalent of each amino acids) and 0.2 M DIPEA (two equivalents of each amino acids). The volume of each coupling reaction was maintained to be 3–5 mL DMF. After drying resins under a vacuum, NOTA-bis(*t*-bu ester) (10 equivalents of primary amine on resin, 100 mg, 0.24 mmol) was manually further coupled to the lysine residue (eight amines per mole loading level, 0.25 mmol/g, 100 mg, 0.025 mmol) on resin with HBTU (89 mg, 0.24 mmol) and DIPEA (83 mg, 0.64 mmol) in DMF (2 mL). NOTA-bis(*t*-bu ester) conjugation was monitored by TNBS assay. When the TNBS test was positive, NOTA-bis(*t*-bu ester) conjugation was performed one more time. After the cleavage of the (NOTA)_8_-NH_2_ mixture from resin in a cocktail of TFA (95%), water (2.5%), and TIPS (2.5%), (NOTA)_8_-NH_2_ (*n* = 2, 13 ± 2 mg, 2.8 μmol) was isolated by HPLC (acetonitrile gradient from 5% to 60% with 0.1%TFA solution for 30 min, retention time: 15.5 min). The mass of (NOTA)_8_-NH_2_was confirmed by MALDI-TOF ([M + H^+^], exact mass was calculated at 4627.62 and found at 4627.20 Da) (Supplementary Fig. 2a).

### Synthesis of (NOTA)_8_-transcyclooctene (TCO)

To a solution of (NOTA)_8_-NH_2_ (1, 3.3 mg, 0.7 μmol) in 1x PBS (1 mL, pH 7.8), transcyclooctene-PEG_4_-NHS (TCO-PEG_4_-NHS, 10 mg, 19 μmol,) dissolved in DMSO (80 μL) was added. pH was readjusted to 8, and the reaction mixture was stirred in a vortex mixer at room temperature for 2–3 h. The solution was diluted with double distilled-water (3–4 mL) and concentrated using a 3 kDa MWCO spin filter unit. Dilution and concentration steps were repeated. (NOTA)_8_-TCO (2, 1 mg, 0.4 μmol) was isolated by HPLC (acetonitrile gradient from 5% to 60% with 0.1%TFA solution for 30 min, retention time: 24.4 min). Mass of (NOTA)_8_-TCO was confirmed by MALDI-TOF (Supplementary Fig. 2a).

### Synthesis of (NOTA)_8_-A555-TCO

(NOTA)_8_-cys(SH)-NH_2_ (4) was similarly synthesized by adding cysteine and a mono-PEG sequence between PEG_27_ and the lysine from (NOTA)_8_-TCO as shown in Supplementary Fig. 2b. After isolation of the product with HPLC, the MALDI-TOF spectrum confirmed the mass of (NOTA)_8_-cys(SH)-NH_2_. (NOTA)_8_-cys(SH)-NH_2_ (2 mg, 0.41 μmol) was reacted with AF555-maleimide (1 mg, 0.79 μmol, Fluoroprobe, Az) in PBS, then the isolation with 3 kDa MWCO spin filter and HPLC afforded (NOTA)_8_-A555-NH_2_ (5, 1 mg, 0.17 μmol). (NOTA)_8_-A555-NH_2_ (1 mg, 0.17 μmol) reacted with TCO-PEG_4_-NHS (3 mg, 5.8 μmol) in 1xPBS (1 mL, pH 8) gave (NOTA)_8_-A555-TCO (6, 350 μg, 0.06 μmol) after HPLC purification (Supplementary Fig. 2b). The AF555, the fluorophore in AF555-MI, is denoted as A555 in the conjugated form.

### Synthesis of (NOTA)_8_-maleimide (MI)

To a solution of (NOTA)_8_-NH_2_ (1, 2 mg, 0.7 μmol) in 1x PBS (0.5 mL, pH 7.4), 0.1 M EDTA (10 μL) and SM(PEG)_2_ (NHS-PEG_2_-MI, 4 mg, 9.4 μmol) dissolved in DMSO (30 μL) were added. The reaction mixture was stirred in a vortex mixer at room temperature for 1 h. The solution was then diluted with 0.05% TFA in water (3–4 mL) and concentrated using a 3 kDa MWCO spin filter unit. Dilution and concentration steps were repeated. (NOTA)_8_-MI (3, 1 mg, 0.2 μmol) was isolated by HPLC (acetonitrile gradient from 5% to 60% with 0.1%TFA solution for 30 min, retention time: 16.6 min). Mass of (NOTA)_8_-MI) was confirmed by MALDI-TOF ([M + H^+^], exact mass was calculated at 4937.74 and found at 4937.66) (Supplementary Fig. 2a).

### Titration and LC-MS/MS

Detailed methods for the titration of single- and multichelators with Cu-63/Cu-64 (shown in Supplementary Fig. 3) and for LC-MS/MS analysis of the Tz-PHP.eB capsid (shown in Supplementary Table 2) are available in the Supplementary Methods section.

### In vitro evaluation of PHP.eB transduction before and after surface amine modification with Tz-NHS ester

To PHP.eB:*CAG-GFP* (6.3 × 10^12^ vg, 10 pmol) in 1x PBS (350 μL, pH adjusted to 8 with 0.1 M Na_2_CO_3_ solution (pH 9.1)), 5 mM tetrazine-PEG_5_-NHS (1 μL, 5 nmol) in anhydrous DMSO was added. After incubation for 1.5 h at room temperature, the reaction mixture was dialyzed in a mini-dialysis device (20 kDa molecular weight cut-off (MWCO)) in 500 mL 1x PBS overnight at 4 °C. The transduction of *CAG-GPF* by Tz-PHP.eB and PHP.eB was compared in HEK293T cells seeded at a density of 70% in a 24-well plate (3×10^4^ cells/well) with 500 μL of Dulbecco’s Modified Eagle’s medium (DMEM; Invitrogen) supplemented with 10% fetal bovine serum (FBS; Invitrogen) and 1% penicillin–streptomycin (Invitrogen) (DMEMc). After incubation at 37 °C and 5% CO_2_ for 24 h, media was aspirated and replaced with fresh DMEMc (250 μL) at 1 × 10^6^ vg/cell of PHP.eB and Tz-PHP.eB. Cells were then incubated at 37 °C in a 5% CO_2_ chamber for 24 h in virus-loaded media, and fluorescent microscopy images (Fig. 2a) were acquired before aspiration and replacement with 500 μL fresh DMEMc. Cells were cultured for one additional day prior to collection. Media was aspirated, and PBS (300 μL) was added. Cells were trypsinized and transferred to 1.5 mL centrifuge tubes. Cells were centrifuged at 1500 rpm for 3 min at room temperature and then washed with 1x PBS. This cell washing cycle was repeated two times. In all, 4% paraformaldehyde (PFA) was added, and the pelleted cells were further kept for 10 min at room temperature to fix the cells. Fixed cells were centrifuged and PFA solution removed. Cells were washed with 1x PBS (500 μL), centrifuged, and supernatant removed. This washing cycle was repeated two times. After cells were resuspended in 1x PBS (500 μL) and transferred to 5 mL flow cytometer tubes on ice, the frequency of green fluorescent cells was analyzed using a flow cytometer (BD FACScan with 488 nm excitation laser) and FlowJo v10.1 software (TreeStar) (Fig. 2b). Methods required for the subsequent evaluation of (NOTA)_8_-PHP.eB transduction (Supplementary Fig. 4) are provided in the Supplementary Methods.

### In vitro evaluation of AAV9 transduction before and after surface amine modification with Tz-NHS ester

AAV9:*CAG-mNeonGreen* (1 × 10^13^ vg, 17 pmol) in 1x PBS (440 μL, pH adjusted to 8 with 0.1 M Na_2_CO_3_ solution (pH 9.1)), 10 mM tetrazine-PEG_5_-NHS (1 μL, 10 nmol) in anhydrous DMSO was added. After reaction and dialysis in 1xPBS, transduction of *CAG-mNeonGreen* by Tz-AAV9 and AAV9 (Fig. 2a, b) was compared in HEK293T cells using the same procedure as above.

### In vitro evaluation of AAV9-TC transduction before and after tetracysteine modification with TCEP

AAV9-TC:*CAG-mNeonGreen* samples (5.8 × 10^12^ vg, 10 pmol) in 1x PBS (100 μL) were incubated for 30 min at room temperature after the addition of 5 mM TCEP (5 nmol, 1 μL). The reaction mixture was dialyzed in a mini-dialysis device (20 kDa molecular weight cut-off (MWCO)) in 500 mL 1x PBS overnight at 4 °C. Transduction of *CAG-mNeonGreen* by HS-AAV9-TC and AAV9-TC (Fig. 2a, b) was compared in HEK293T cells using the same procedure as above. Methods required for the subsequent evaluation of (NOTA)_8_-AAV9-TC transduction (Supplementary Fig. 5) are provided in the Supplementary Methods.

### Sodium dodecyl sulfate—polyacrylamide gel electrophoresis of AAVs

AAVs used for in vitro transduction and in vivo PET studies were concentrated to 20 μL and 150 μL volume, respectively, using a spin filter (MWCO 100k). AAV capsids (5–10 μL) were denatured in tris-glycine Sodium dodecyl sulfate (SDS) sample buffer (ThermoFisher Scientific, LC2676) and treated for 2 min at 85 °C. This solution was loaded into and separated on a 14% tris-glycine mini gel (ThermoFisher Scientific, XP00140BOX) under 225 V for 45 min in an electrophoresis chamber filled with tris-glycine SDS running buffer (ThermoFisher Scientific, LC2675). Sharp pre-stained protein standard ladder protein images (ThermoFisher Scientific, LC5800) were loaded to compare the molecular weight of viral proteins. The gel was washed with double distilled water, stained with Coomassie staining solution (ThermoFisher Scientific, LC6060) and imaged using an iPhone 6 cellular phone camera (Fig. 2e, Supplementary Figs. 4b and 5b). The gels loaded with radiolabeled AAVs were further exposed on a phosphor screen in a cassette (Molecular Dynamics, CA) overnight, which was then scanned using the Phosphorimager (Amersham Bio-Science, NJ). For polyacrylamide gel electrophoresis (PAGE) of AAVs in Supplementary Figs. 4b and 5b, the same procedure was performed without gel autoradiography.

### In vivo transduction assay with ^63^Cu-PHP.eB and PHP.eB

The synthesis of Cu-PHP.eB was performed by the following procedure. Tetrazine-PEG_5_-NHS in anhydrous DMSO (1 μL, 0.5 mM) was added to PHP.eB:*CAG-GFP* (2.1 × 10^12^ vg, 3.5 pmol) in 1x PBS (180 μL, pH adjusted to 8 with 0.1 M Na_2_CO_3_ solution (pH 9.1)). After incubation for 1 h at room temperature, the reaction mixture was dialyzed in a mini-dialysis device (20 kDa molecular weight cut-off (MWCO)) at 4 °C overnight. The dialyzed solution was incubated with copper-incorporated (NOTA)_8_-TCO (5 μL, 10 μM) for 30 min and then diluted to 15 mL with PBS. The solution was concentrated using a centrifugal filter unit (MWCO 100k, 3000×*g* for 10 min). Dilution in 15 mL PBS and concentration was repeated twice. The size of the isolated ^63^Cu-PHP.eB and non-labeled PHP.eB was measured using a Zetasizer (Malvern). After titration of the ^63^Cu-PHP.eB and the non-labeled PHP.eB solution, 1.5 × 10^10^ vg of ^63^Cu-PHP.eB:*CAG-GFP* or PHP.eB:*CAG-GFP* as a control were injected in C57BL/6 mice (*n* = 2 per group). After three weeks, mice were euthanized by Euthosol under deep isoflurane and perfused with DMEM followed by 4% paraformaldehyde (in PBS). The harvested brains were further fixed in 4% paraformaldehyde (PFA) in PBS for a day. Fluorescent images of the sliced brain were aquired and quantified (Fig. 2c, d).

### Radiolabeling of AAVs

All radiolabeling experiments were conducted under a radiation use authorization approved by the University of California, Davis (Davis, CA) and Stanford University (Palo Alto, CA). Molar activity in Fig. 1 is calculated from the average molar activity described in the quality control report from the Washington University, School of Medicine MIR cyclotron facility (the source of Cu-64 used in this study). For radiolabeling of AAV9 and PHP.eB, pH of AAV9:*CAG-mNeonGreen* (1 × 10^13^ vg, 16 pmol in 0.4 mL PBS) and PHP.eB:*CAG-GFP* (4.2 × 10^12^ vg, 7 pmol in 0.2 mL PBS) was adjusted to 8 with 0.1 M Na_2_CO_3_ solution (pH 9.2), and 1 mM tetrazine-PEG_5_-NHS (1 nmol, 1 μL) in anhydrous DMSO was added to the AAV solutions. The reaction mixtures were incubated at 4 °C for 2 h, transferred to a mini-dialysis device (20 kDa molecular weight cut-off (MWCO)), and dialyzed in 1x PBS (0.5 L) overnight. Tetrazine-conjugated AAVs (Tz-AAVs) were further reacted with ^64^Cu-chelated (NOTA)_8_-TCO for 30 min, freshly prepared from a reaction of ^64^CuCl_2_ (37-74 MBq (1–2 mCi), 2–3 μL) and 10 μM (NOTA)_8_-TCO (20 pmol, 2 μL) in ammonium citrate buffer (10 μL, pH 6.5). The incorporation of Cu-64 to (NOTA)_8_-TCO was monitored by instant thin-layer chromatography and completed in 30 min. Radiolabeled AAVs (^64^Cu-AAV9 and -PHP.eB) were purified by size-exclusion chromatography (bed volume: 3 mL) or centrifugal filter unit (MWCO 100k, 3000 g for 10 min) with three cycles of dilution with PBS (15 mL) and concentrated in ~200 μL volume. For radiolabeling of AAV9-TC, AAV9-TC:*CAG-mNeonGreen* (3 × 10^13^ vg, 50 pmol, in 0.1 mL PBS) was treated with 5 mM tris(2-carboxyethyl)phosphine (TCEP) (5 nmol, 1 μL) for 30 min. ^64^Cu-chelated (NOTA)_8_-MI, obtained from the incorporation of ^64^CuCl_2_ (74 MBq (2 mCi), 2.5 μL) to 10 μM (NOTA)_8_-MI (35 pmol, 3.5 μL) in ammonium citrate buffer (10 μL, pH 6.5), was added to disulfide-reduced SH-AAV9-TC and then the pH adjusted to 7–7.5 with 0.1 M NaOH. After incubation at room temperature for 1 h, ^64^Cu-AAV9-TC was isolated by size-exclusion chromatography and concentrated in ~200 μL volume.

### PHP.eB and AAV9 labeling with (NOTA)_*8*_-A555-TCO for the characterization of the number of labels per AAV particle

PHP.eB:*CAG-mNeonGreen* (1.2 × 10^12^ vg, 2 pmol) or AA9:*CAG-mNeonGreen* (2 × 10^12^ vg, 3.3 pmol) in PBS buffer (pH 8, 0.1 mL) was reacted with tetrazine-PEG_5_-NHS (0.4–0.7 and 0.7–1 equivalents, respectively) at room temperature for 30 min. After addition of 0.1 mL PBS, the reaction mixture was dialyzed (MWCO 20k) in PBS buffer three times (15 mL each dialysis for 2 h and 50 mL overnight). Dialyzed Tz-PHP.eB or -AAV9 mixtures were reacted with (NOTA)_8_-A555-TCO (20-40 and 30-50 pmol, respectively) for 30 min at room temperature. Reaction mixture was diluted to 15 mL with PBS and filtered in a centrifugal filter unit (MWCO 100k, 3000×*g* for 10 min). To the concentrated product in PBS (~0.15 mL), PBS (15 mL) was added and centrifuged (MWCO 100k, 3000×*g* for 10 min). The concentrated solution (~0.15 mL) was transferred to a centrifugal tube, and the volume was adjusted to 0.2 mL. The number of AAVs and labels were then measured (Supplementary Table 3).

### AAV9-TC labeling with A555-C2-MI for the characterization of the number of labels per AAV particle

AAV9-TC:*CAG-mNeonGreen* (1.7 × 10^12^ vg, 3 pmol) in PBS (pH 7, 0.1 mL) was treated with 0.1 mM tris(2-carboxyethyl)phosphine (TCEP) (0.3 nmol) for 30 min. Alexa555-C2-MI (60 pmol) in dissolved in anhydrous DMSO was added to a reduced form of AAV9-TC (SH-AAV9-TC). After incubation at room temperature for 1 h, the reaction mixture was diluted with 15 mL PBS and filtered in a centrifugal filter unit (MWCO 100k, 3000×*g* for 10 min). To a concentrated product in PBS (~0.15 mL), PBS was added again and centrifuged (MWCO 100k, 3000×*g* for 10 min). Concentrated solution (~0.15 mL) was transferred to a centrifugal tube, and the volume was adjusted to 0.2 mL. The number of AAVs and labels were then measured. The resulting optical label is denoted as A555 at Supplementary Table 3.

### PHP.eB and AAV9 labeling with A555 for in vivo optical imaging

PPHP.eB:*CAG-DIO-GFP* and AAV9:*CAG-mNeonGreen* was directly labeled with Axexa555-NHS ester. In brief, 1 mM Alexa555-NHS (2 μL in DMSO, 2 nmol) was added to PHP.eB (7 × 10^12^ vg, 12 pmol) or AA9 (5.2 × 10^12^ vg, 7 pmol) in PBS buffer (pH 8). After 1 h, the mixture was transferred to a mini-dialysis device (20 kDa molecular weight cut-off (MWCO) and dialyzed in 1xPBS (0.5 L) overnight. The dialyzed solution was diluted with 15 mL of PBS solution and filtered in a centrifugal filter unit (MWCO 100k, 3000×*g* for 10 min) with three cycles of dilution with PBS (15 mL). This solution was reformulated with 1x PBS before the in vivo study shown in Supplementary Fig. 9. The resulting label is denoted as A555 in Supplementary Figs. 7a and 9.

### PHP.eB and AAV9 labeling with (NOTA)_*8*_-A555-TCO for in vivo optical imaging

PHP.eB:*CAG-DIO-GFP* and AAV9:*CAG-mNeonGreen* was labeled with tetrazine-PEG_5_-NHS using the same procedure as above. In brief, 1 mM tetrazine-PEG_5_-NHS (2 μL in DMSO, 2 nmol) was added to PHP.eB (1.4 × 10^13^ vg, 23 pmol) in PBS buffer (pH 8). After 1 h, the mixture was transferred to a mini-dialysis device (20 kDa molecular weight cut-off (MWCO)) and dialyzed in 1x PBS (0.5 L) overnight. Tz-PHP.eB solution was reacted with 0.1 mM (NOTA)_8_-A555-TCO (1 μL, 0.1 nmol) for 30 min at room temperature. The reaction mixture was diluted to 15 mL PBS solution and filtered in a centrifugal filter unit (MWCO 100k, 3000 g for 10 min) with three cycles of dilution with PBS (15 mL). Concentrated (NOTA)_8_-A555-PHP.eB was formulated with 1x PBS for the in vivo study shown in Fig. 3i and Supplementary Fig. 10.

### Animal models

All animal experiments were conducted under an animal use protocol approved by the University of California, Davis, Institutional Animal Care and Use Committee (IACUC) or Stanford University, Administrative Panel on Laboratory Animal Care (APLAC). For PET studies and biodistribution, ^64^Cu-labeled AAVs were evaluated in wild-type 7–9-week-old female C57BL/6 and 9 week-old-female BALB/c mice (The Jackson laboratory, Bar Harbor, ME). For neuraminidase-treated BALB/c mice, neuraminidase (0.12 units/20 μL, Sigma-Aldrich, #N7885) was ﻿intranasally administered 3 h before the injection of ^64^Cu-AAVs. The classical pharmacokinetics and biodistribution studies with qPCR were conducted with wild-type 5–6 week-old female C57BL/6 mice (Charles River).

### PET/CT scans and biodistribution

Mice were anesthetized with 3.0% isoflurane in oxygen and maintained under 1.5–2.0% isoflurane. ^64^Cu-AAV9 injections in C57BL/6 mice (*n* = 3, 421 ± 25 KBq), ^64^Cu-AAV9-TCO injections in C57BL/6 mice (*n* = 3, 760 ± 156 KBq), ^64^Cu-PHP.eB injections in C57BL/6 mice (*n* = 3, 628 ± 581 KBq), BALB/c mice (*n* = 3, 477 ± 19 KBq), or BALB/c mice treated with neuraminidase (*n* = 3, 718 ± 30. KBq) were administered via the tail vein on an Inveon DPET small animal PET scanner (Siemens Medical Solutions USA, Knoxville, TN). Animals were scanned for 30 min at 0, 4, and 21 h post injection. After each PET scan, the animals were moved to a small animal Inveon MM CT system (Siemens Medical Solutions USA, Knoxville, TN) and a CT scan was conducted to obtain anatomical information for co-registration of PET/CT images. After the final imaging time point, mice were euthanized by Euthosol under deep isoflurane. Animals were then perfused with DMEM solution. Blood, heart, lungs, liver, spleen, kidneys, stomach, intestine, muscle, bone, and brain were harvested for biodistribution analysis. Radioactivity in each organ was measured with a 1470 automatic gamma counter (PerkinElmer, CT) after which organ weights were taken on a balance. Biodistribution of AAVs was presented as percent injected dose per gram (% ID/g). In some cases, brains were sectioned at 2 mm thickness to obtain ex vivo autoradiography.

### ROI analysis and time–activity curves

All PET images were reconstructed with the maximum a posteriori (MAP) reconstruction algorithm in Inveon Acquisition Workspace (Siemens Medical Solutions Inc., USA) and analyzed with Inveon Research Workspace 4.2 (Siemens Medical Solutions Inc., USA) after the co-registration of PET/CT images. Regions of interest (ROIs) were drawn in the heart chamber for blood, whole brain, and liver. The time-activity curves (TAC) of blood, brain, and liver radioactivities at 0, 4, and 21 h were analyzed with Prism 8 (Graphpad). Blood TACs were fitted with one phase decay. Radioactivity density from image analysis is presented as percent injected dose per cubic centimeter (% ID/cc).

### Early brain accumulation and Logan analysis of ^64^Cu-AAVs

Initial PET data from the 30 min scan was segmented into six frames (5 min/frame). Blood radioactivity in the brain (% ID/cc) over time was calculated by multiplying the brain radioactivity by the brain vascular volume (8%) (estimated by the ratio of brain and blood radioactivity of AAV9 and AAV9-TC in C57BL/6 and PHP.eB in BALB/c at 5 min after injection). The blood radioactivity over time was then subtracted from total brain activity (% ID/cc) to estimate the brain accumulation. A Logan plot (Fig. 3h) was then applied for the calculation of the uptake rate of each AAV in the brain at a given time after the administration of AAVs^17^. C_B_(t) and C_T_(t) are the radioactivity concentration in the blood and target at a given time, and IntC_B_(t) and IntC_T_(t) are the accumulated radioactivity in the blood and target, respectively, from the time of injection to time (t). IntC_T_(t)/C_T_(t) vs IntC_B_(t)/C_T_(t) was then plotted.

### Confocal microscopy

For imaging of fluorescent AAVs (Fig. 3i and Supplementary Fig. 9), mice were anesthetized with 3.0% isoflurane in oxygen and maintained under 1.5–2.0% isoflurane. A555-PHP.eB (0.1 mL, 1 × 10^11^ vg) or (NOTA)_8_-A555-PHP.eB (0.1 mL, 2 × 10^10^ vg) or was administered to C57BL/6 mice (*n* = 14 total) through tail vein injection. Mice were euthanized at 4, 24 and 48 h by Euthosol under deep isoflurane, and perfused with DMEM solution followed by 4% PFA in PBS at pH 7. To stain the brain endothelium, FITC-lectin (25 μL, 50 μg) was injected via the tail-vein 15 min before euthanization. Brains were collected and kept in 4% PFA overnight. Brain tissue was then sliced to 100 μm thick sections on a vibratome (Leica VT-1000S) in PBS and mounted onto microscope slides. Fluorescence images of AAV distribution on the endothelium were acquired with a confocal microscope (Zeiss AxioObserver), a ×40 objective, 488 and 561 nm lasers, and HQ camera. The microscope was controlled by Slidebook software (Intelligent Imaging Innovations).

Brain slices for in vivo transduction (Figs. 2c and 3g) were mounted on microscope slides and imaged using a confocal microscope (Leica DMi8) controlled with LAS X software. All images were recorded at the same laser power and gain control. Images were acquired with a 10x lens and fluorescence images were analyzed using ImageJ.

### Synthesis of PEG(40  kDa)-AAV9 for cryo-electron microscopy

To a solution (0.4 mL, PBS) of PEG(40 kDa)-NH_2_ (2.5 mg, 62 nmol) was added TCO-PEG_4_-NHS ester (0.5 mg, 0.86 μmol). The pH was adjusted to 8.6 with 0.1 M sodium carbonate (pH 9.2). After a 2 h reaction at room temperature, the reaction mixture was diluted with 1 mL deionized water then dialyzed overnight with 10k MWCO and the purified solution was transferred to a cryotube and freeze-dried (Supplementary Fig. 7b). PEG(40 kDa)-AAV9 was obtained as illustrated in Supplementary Fig. 7a. Tz-AAV9 was obtained from a reaction with AA9:*CAG-mNeonGreen* (5.9 × 10^12^ vg, 9.8 pmol) and tetrazine-PEG_5_-NHS (2 μL in DMSO, 2 nmol) under the same procedure as in Optical labeling of AAVs and was reacted with 10 μM PEG (40 kDa)-TCO (4 μL, 40 pmol) for 30 min at room temperature. The reaction mixture was diluted to 15 mL PBS solution and filtered in a centrifugal filter unit (MWCO 100k, 3000 g for 10 min) with two cycles of dilution with PBS (15 mL). PEG (40 kDa)-AAV9 was further concentrated to 40 μL volume for cryo-EM.

### Cryo-electron microscopy of PEG(40  kDa)-AAV9

Cryo-Electron microscopy collection was performed on a Glacios™ Cryo-TEM operating with a field-emission gun at 200 kV. Cryo-EM grid was prepared with a 30 μL solution containing PEG(40 kDa)-AAV9, which was placed on Quantifoil R1/2 Cu 300 mesh grids. The grids were pretreated with 10 mAmp of glow discharge for 40 seconds. After 1 min of on-grid incubation the excess solution was removed and quickly plunged into liquid ethane using an FEI Vitrobot Mark III semi-automated cryo-plunger. The PEG(40 kDa)-AAV9 particles were embedded into a thin layer of vitrified ice and transferred into the imaging chamber using a Gatan 626 cryo-transferring system. The grids were examined at ×50,000 magnification and images were captured using a Gatan K3. The cryoEM images were inverted in contrast to illustrate the positive density of the molecular mass against the dark ice background. The digital images were recorded with a pixel size of 0.85 Å using autofocusing scripts in the Serial-EM package set to a defocus range of 0.5–2.0 micron. The digital images with minimum stigmatism or drift were selected for further statistical analysis and figure preparation. Corresponding EM images are shown in Fig. 2g.

### Biodistribution and pharmacokinetics of AAVs with qPCR

For the biodistribution study in Fig. 3f, g, PHP.eB (7.4 × 10^10^ vg, *n* = 4) and (NOTA)_8_-A555-PHP.eB (1.4 × 10^11^ vg, *n* = 4) in PBS (0.1 mL) were injected through the mouse (C57BL/6) tail vein. At 21 h after injection, mice were euthanized by Euthosol under deep isoflurane, and then perfused with DMEM. Heart, lungs, liver, spleen, kidneys, brain, and blood were harvested as 3–4 fractions per tissue and 100–200 μL blood per mouse. Collected tissues were immediately frozen in 2 mL cryotubes under liquid nitrogen and stored at −80 °C. Viral vector and mouse genomic DNAs were extracted from tissues by a DNeasy Blood and Tissue Kit (Qiagen) according to the kit protocol and quantified by quantitative polymerase chain reaction (qPCR) with a TaqMan assay. The primers and TaqMan probe used for the WPRE sequence were 5′-GCATTGCCACCACCTGTCA (forward) and 5′-TCCGCCGTGGCAATAGG (reverse), and 5′-CTTTCCGGGACTTTCG (FAM). The primers and TaqMan probe targeting the mouse glucagon gene as a housekeeping gene were 5′-GTTCTCTCTGTATTGTCCTTTCAAAGTCT (forward) and 5′-CAAAGTCCCTGAAGGTTCTGAGATG (reverse), and 5′-CCCTGGTCATGTTTTTAA (FAM). Quantification was performed by a standard curve from a serial dilution of known standard plasmid concentration. For the classical PK study of blood clearance, PHP.eB (1.35 × 10^11^ vg, *n* = 4) and (NOTA)_8_-A555-PHP.eB (1.17 × 10^11^ vg, *n* = 4) in PBS (0.1 mL) were injected through the mouse (C57BL/6) tail vein. Blood (~100–200 μL) was collected from the mouse orbital sinus at 0.5, 4, and 21 h after administration to a Microtrainer containing EDTA (BD science, NJ). Collected blood was immediately transferred to 2 mL cryotubes (Sarstedt, Germany) and further frozen in liquid nitrogen. The viral vectors from 100 μL of blood were extracted by DNeasy Blood and Tissue Kit (Qiagen) according to the kit protocol and quantified by the TaqMan assay targeting the WPRE sequence. qPCR was performed with QuantStudio6 Flex (Applied Biosystems) and CFX96 Touch real-time PCR detection system (Bio-Rad).

### Image processing and data analysis

Microscopic image process and ROI analyses were performed using ImageJ, LAS X (Leica) and SlidBook6 (3i). For PET/CT data and image processing, Inveon Acquisition Workspace (Siemens) and Inveon Research Workspace 4.2 (Siemens) was used, respectively. Microsoft Excel (ver. 16.35), and GraphPad Prism 8 for macOS were used for general data and statistical analysis. PyMOL 2.0 (Molecular Graphics System) was used to process the structure of capsid. FlowJo v10.1 (Treestar) was used for data analyses of results from flow cytometry. Gating/sorting strategy is presented at Supplementary Fig. 11 and reporting summary.

## Statistics and reproducibility

All statistical analyses were performed in GraphPad Prism software (Prism 8.0). The statistical tests with confidence intervals, effect sizes, degree of freedom and *P* values can be found in the source data. Sample size for each experiment is shown in the appropriate caption and the biological replicates across experiments are summarized below. Cell experiments in Fig. 2a, b were repeated 4, 3, and 1 times for PHP.eB, AAV9 and AAV9-TC. For Fig. 2c, fluorescent images were selected from multiple mouse brain slices obtained in 2 experiments. For Fig. 2g, the cryoEM images result from a single experiment. For Fig. 3a–e and G-H, PET/CT and autoradiography result from two experiments. For Fig. 3f, qPCR results were obtained from one set of experiments. For Fig. 3i, representative fluorescence images with the multichelator AAV were chosen from mice euthanized at 4 (two experiments), 24 (one experiment) and 48 h (one experiment). For Fig. 4, the data result from one series of experiments.

### Reporting summary

Further information on research design is available in the Nature Research Reporting Summary linked to this article.

## Supplementary information

Supplementary Information

Description of Additional Supplementary Files

Supplementary Movie 1a

Supplementary Movie 1b

Supplementary Movie 2a

Supplementary Movie 2b

Supplementary Movie 3a

Supplementary Movie 3b

Supplementary Movie 4a

Supplementary Movie 4b

Reporting Summary

## Data Availability

The raw data files from mass spectrometer were processed using Byonic v 2.14.27 (Protein Metrics, San Carlos, CA) to identify peptides and subsequently infer proteins using the *Mus musculus* database from the Universal Protein Resource (UniProt, http://www.uniprot.org) along with the sequences of capsid proteins. Protein Data Bank (PDB ID:3Ux1) was used to display capsid structure. Analyzed viral protein sequence data are available in the source data. The authors declare that image and quantitative data supporting the findings of this study are available within the paper and the source data and supplementary files. The raw PET images and associated data that support the findings of this study are available from the corresponding author upon reasonable request.

## Source data

Source Data
