# Supplementary material for: Positron emission tomography imaging of novel AAV capsids maps rapid brain accumulation
Source: Nat Commun. 2020 Apr 30;11:2102. doi: 10.1038/s41467-020-15818-4 (PMC7193641; doi:10.1038/s41467-020-15818-4)
Supplement: Supplementary file 1 — Supplementary Information [file 41467_2020_15818_MOESM1_ESM.pdf]

Supplementary information

Positron emission tomography imaging of novel AAV capsids maps rapid brain accumulation

J. W. Seo et al.

Contents:

Supplementary Methods
Supplementary Tables 1-11
Supplementary Figures 1-11
Supplementary References

Supplementary methods

Materials and reagents for multichelator synthesis and AAV labeling. Rank Amide-Chemmatrix® resin (Biotage®, 7-600-1310-25), HBTU (Novabiochem, 851006, *N,N,N′,N′*-tetramethyl-O-(1H-benzotriazol-1-yl)uronium hexafluorophosphate, O-(benzotriazol-1-yl)-*N,N,N′,N′*-tetramethyluronium hexafluorophosphate), DIPEA (Sigma-Aldrich, 496219, *N,N*-diisopropylethylamine)
TIPS (Sigma-Aldrich, 233781, triisopropylsilane), Fmoc-PEG_{27}-OH (Polypure, 15137-2790), Fmoc-PEG_3-OH (Chem-Impex International Inc.,07310), 4-methylpiperidine (Acros, 127515000, 99%) TFA (Acros, 13972, trifluoroacetic acid), NOTA-bis(t-Bu ester)(Macrocyclics, B-620,1,4,7-triazacyclononane-1,4-bis-tert-butyl acetate-7-acetic acid), tetrazine-PEG_5-NHS ester (Click Chemistry Tools, 1143), transcyclooctene-PEG_4-NHS ester: (Click Chemistry Tools, A137), $SM(PEG)_2$ (ThermoFisher Scientific, 22102, succinimidyl-[(*N*-maleimidopropionamido)-diethyleneglycol] ester). Alexa555-NHS (ThermoFisher Scientific, A20009), AF555-maleimide (Fluoroprobes, 1168). Alexa555-C2-maleimide (ThermoFisher Scientific, A20346).

Preparation of AAV9, AAV9-TC, and PHP.eB. Tetracysteine motif HRWCCPGCCKTF was cloned into an AAV9 backbone (tTA-iCAP-AAV9) using overlap PCR into position 139 of AAV9 capsid region (*AAV9-TC-forward:* CATCGATGGTGTTGCCCGGGCTGCTGTAAGACTTTCgctcctggaaagaagaggcctgtag and *AAV9-TC-reverse:* GAAAGTCTTACAGCAGCCCGGGCAACACCATCGATGcgtcttagccgcttcctcaaccaga). AAV9-TC and AAV9 packaging *CAG-mNeonGreen* were produced following protocol outlined in Challis, et al[1]. Briefly, HEK293T cells (ATCC) were triple transfected using polyethylenimine (PEI); virus was collected after 120 h from both cell lysates and media and purified over iodixanol (Optiprep, Sigma) via ultracentrifugation. Maleimide labelling of AAV9-TC was confirmed per methods described in Chandran, el al[2]. PHP.eB packaging *CAG-mNeonGreen* or *CAG-DIO-GFP* were also prepared similar to above.

Titration of single- and multichelators with Cu-63/Cu-64 for Supplementary Fig. 3. A 20 pmol of single chelator in double distilled water (2 μL) was added to two-fold serial dilutions of $CuCl_2$ solution (3 μL) from 0.01 mM to 0.3 nM, spiked with 0.33 pmol of $^{64}CuCl_2$ (1 μL). The titration of 20 pmol of multichelator was performed with $CuCl_2$ solution (3 μL) from 0.1 mM to 0.3 nM, spiked with 14 pmol of $^{64}CuCl_2$ (1 μL). After 30 min incubation of reaction mixtures at room temperature, incorporation yields were confirmed by ITLC. The ratio of Cu/chelator was plotted over incorporation yield as shown in Supplementary Fig. 3.

***In vitro* transduction assay for $(NOTA)_8$-PHP.eB for Supplementary Fig. 4,5.** Evaluation of PHP.eB:CAG-GFP transduction after multichelator conjugation was performed based on the following methods. To each PHP.eB:*CAG-GFP* ($4.2x10^{11}$ vg) in 1xPBS (180 μL, pH adjusted to 8 with 0.1 M Na_2CO_3 solution (pH 9.1)), 0.7 μL of 1, 5, or 10 mM tetrazine-PEG_5-NHS in anhydrous DMSO was added. After incubation for 1 hour at room temperature, each reaction mixture was dialyzed in a mini-dialysis device (20 kDa molecular weight cut-off (MWCO)) at 4 ºC for 6 h. To each reaction, 0.7 μL of 0.1, 0.5, or 1 mM $(NOTA)_8$-PEG_{27}-TCO in 1xPBS was added, respectively. The reaction mixture was dialyzed overnight at 4 ºC. Purified AAVs were transferred to microcentrifuge tubes, and the volume of each AAV solution was adjusted to 300 μL with 1xPBS as shown in Supplementary Fig. 4A. The transduction of the *CAG-GFP* gene by Tz-PHP.eB and PHP.eB were compared in HEK293T cells seeded at a density of 70% in a 24-well plate ($3x10^4$ cells/well) with 500 μL of Dulbecco's Modified Eagle's medium (DMEM; Invitrogen) supplemented with 10% fetal bovine serum (FBS; Invitrogen) and 1% penicillin–streptomycin (Invitrogen) (DMEMc). After incubation at 37 °C and 5% CO_2 for 24 h, media was aspirated and replaced with fresh DMEMc (250 μL) at $1x10^6$ vg/cell of unmodified control (UMC, n=4) PHP.eB or modified PHP.eB with $(NOTA)_8$-PEG_{27}-TCO in the following ratios (100, 500, and 1000 $(NOTA)_8$-PEG_{27}-TCO/AAV ratio, n=4 for each ratio). Cells were then incubated at 37 °C in a 5% CO_2 chamber overnight in virus-loaded media before aspiration and replacement with 500 μL fresh DMEMc. Cells were cultured for one additional day prior to collection. Media was aspirated, and PBS (300 μL) was added. Cells were trypsinized and

transferred to 1.5 mL centrifuge tubes. Cells were centrifuged at 1500 rpm for 3 minutes at room temperature and then washed with 1xPBS. This cell washing cycle was repeated two times. 4% paraformaldehyde (PFA) was added, and the pelleted cells were further kept for 10 min at room temperature to fix the cells. Fixed cells were centrifuged and PFA solution removed. Cells were washed with 1xPBS (500 μL), centrifuged, and supernatant was removed. This washing cycle was repeated two times. After cells were resuspended in 1xPBS (500 μL) and transferred to 5 mL flow cytometer tubes on ice, the frequency of cells with green fluorescence was analyzed using a flow cytometer (BD FACScan). All flow cytometry data were analyzed using FlowJo v10.1 software (TreeStar).

***In vitro t*ransduction assay for $(NOTA)_8$-AAV9-TC before and after tetracysteine modification.** AAV9-TC:*CAG-mNeonGreen* samples ($5.8x10^{12}$ vg, 10 pmol in 1xPBS (100 μL) were incubated for 30 minutes after the addition of 5 mM TCEP (5 nmol, 1 μL). To each reaction, 1 μL of PBS only, 0.1, 0.5, or 1 mM $(NOTA)_8$-PEG_5-MI (0.1, 0.5, and 1 nmol) in 1xPBS was added. After incubation for 1 h at room temperature, each reaction mixture was filtered through a spin column (Bio-rad, MWCO 6k) and collected into centrifuge tubes. For the transduction assay, untreated control PHP.eB was compared to the dialyzed AAVs as shown in Supplementary Fig. 5A. HEK293T cells were seeded at a density of 70% in 24-well plates ($3x10^4$ cells/well) with 500 μL of DMEMc (Invitrogen). After incubation for 24 h, media was aspirated and replaced fresh DMEMc (250 μL) at $1x10^6$ vg/cells of unmodified control (n=4) AAV or treated AAV9-TC with $(NOTA)_8$-PEG_{27}-MI in the following ratios (14, 70, and 140 multichelator/AAV ratio, n=4 for each ratio). Cells were then incubated at 37 °C in a humidified CO_2 chamber overnight in virus-loaded media before aspiration and replacement with 500 μL fresh DMEM. Cells were cultured for one additional day prior to collection, fixation and analysis of cellular mNeonGreen expression, which was done using a flow cytometer (BD FACScan) as above.

LC-MS/MS analysis of Tz-PHP.eB capsid.
For mass analysis of modified capsid protein, Tz-PHP.eB capsid were denatured with SDS-sample buffer (Thermo Fisher Scientific, San Jose, CA) after incubation for 2 min at 85°C. Denatured sample solution was run on a 12% tris-glycine gel (Thermo Fisher Scientific, San Jose, CA) in tris-glycine running buffer (Thermo Fisher Scientific, San Jose, CA) and resolved by Coomassie staining. Protein gel bands were excised out into 1.5 ml Eppendorf tubes and further cut into 1x1 mm squares. The excised gel pieces were then reduced with 5 mM DTT in the presence of 50 mM ammonium bicarbonate at 55°C for 30 min. Residual solvent was removed and alkylation was performed using 10 mM acrylamide in 50 mM ammonium bicarbonate for 30 min at room temperature. The gel pieces were rinsed 2 times with 50% acetonitrile, 50 mM ammonium bicarbonate and placed in a speed vacuum for 5 min. Digestion was performed with Trypsin/LysC (Promega) in the presence of 0.02% protease max (Promega) in a standard overnight digest at 37°C. Samples were then centrifuged and the solvent including peptides was collected. Further peptide extraction was performed by the addition of 60% acetonitrile, 39.9% water, 0.1% formic acid and incubation for 10-15 min. The peptide pools were dried in a speed vac. Samples were reconstituted in 12.5 μl reconstitution buffer (2% acetonitrile with 0.1% formic acid) and 3 μl of each sample were injected into the instrument.

For mass spectrometry, experiments were performed using an Orbitrap Fusion Tribrid mass spectrometer (Thermo Scientific, San Jose, CA) with liquid chromatography using a Nanoacquity UPLC (Waters Corporation, Milford, MA). For a typical LCMS experiment, a flow rate of 450 nL/min was used, where mobile phase A was 0.2% formic acid in water and mobile phase B was 0.2% formic acid in acetonitrile. Analytical columns were prepared in-house with an inner diameter of 100 microns pulled to a nanospray emitter using a P2000 laser puller (Sutter Instrument, Novato, CA). The column was packed using C18 reprosil Pur 1.8 micron stationary phase (Dr. Maisch) to a length of ~25 cm. Peptides were directly injected onto the analytical column using a gradient (2-45% B, followed by a high-B wash) of 80min. The mass spectrometer was operated in a data dependent fashion using a combination of ETD in Ion trap and HCD in Orbitrap fragmentation for MS/MS spectra generation.

The raw data files were processed using Byonic v 2.14.27 (Protein Metrics, San Carlos, CA) to identify peptides and subsequently infer proteins using the *Mus musculus* database from the Universal Protein Resource (UniProt, http://www.uniprot.org) along with the sequences of capsid proteins. Proteolysis with trypsin/lys-C mix (0.125 ng, mass grade, Promega corporation) was assumed to be semi-specific allowing for N-ragged cleavage with up to two missed cleavage sites. Precursor and fragment mass accuracies were held within 12 ppm. Proteins were held to a false discovery rate of 1%, using standard approaches.

Supplementary Tables

Supplementary Table 1. Summary of AAV labeling conditions from previous literature

Virus	Amount (volume used)	NHS-ester	Reaction ratio (dye:AAV)	Resulting dye/AAV ratio	buffer	Reaction time	Isolation	Ref
AAV	1.33×10^{14} particles/mL (1 mg/mL)	Cy3- or Cy2-NHS	NS	1.6-2.3	0.1 M Na_2CO_2 $NaHCO_3$ (pH 9.3)	30 min	Dialysis	[3,4]
AAV2	1×10^{10} particles	Alexa488-TFP	3×10^{6}:1	NS	0.1 M $NaHCO_3$ (pH 9.3)	2 h	Dialysis	[5]
AAV2	5×10^{11} particles (1 mL)	Cy3-NHS	NS	1	0.1 M $NaCO_3$ (pH 9.3)	30 min	Dialysis	[6]
AAV2	-	Alexa488-NHS	-	2	-	-	Dialysis	[7]
AAV	10^{12} vg/mL (2 μL used)	SPA-PEG (20ul total volume)	1-1000 (PEG/lysine)		HEPES (pH7.4)	3 h	-	[8]
NS: not-specified								

Supplementary Table 2. Protein sequence alignment of serotype AAVs compared to PHP.eB. Red represents the labeled lysine (K) within adjacent amino acids, and black is the corresponding lysine (K) or arginine (R) site located in a similar sequence in serotype AAV1-10. The most frequently modified lysines in PHP.eB were those shown in the 4th and 5th columns.

	Frequency of modified lysines in mass list of each viral protein						
PHP.eB	NGLDKGEPVN (57-66)	NPYLKYNHAD (88-97)	MASHKEGEDR (524-533)	VDADKVMITN (553-562)	EEEIKTTNPV (563-572)	PIWAKIPHTD (614-623)	ELQKENSKRW (693-702)
VP1	1	1	-	13	8	-	-
VP2	-	-	3	14	10	-	1
VP3	-	-	4	17	11	3	1
	**Sequence alignment of serotype AAVs with PHP.eB in modified lysine sites**						
AAV1	NGLD**K**GEPVN (57-66)	NPYL**R**YNHAD (88-97)	MASH**K**DDEDK (524-533)	TALDNVMITD (553-562)	EEEI**K**ATNPV (563-572)	PIWA**K**IPHTD (614-623)	ELQ**K**ENS**K**RW (686-695)
AAV2	NGLD**K**GEPVN (57-66)	NPYL**K**YNHAD (88-97)	MASH**K**DDEEK (523-532)	VDIE**K**VMITD (552-559)	EEEI**R**TTNPV (562-571)	PIWA**K**IPHTD (616-625)	ELQ**K**ENS**K**RW (685-684)
AAV3	NGLD**K**GEPVN (57-66)	NPYL**K**YNHAD (88-97)	MASH**K**DDEEK (524-533)	AELDNVMITD (553-562)	EEEI**R**TTNPV (563-572)	PIWA**K**IPHTD (617-626)	ELQ**K**ENS**K**RW (686-695)
AAV4	NGLD**K**GEPVN (56-65)	NPYL**K**YNHAD (87-96)	MATAGPADSK (523-532)	TVPGTLIFTS (551-560)	EEELAATNAT (561-70)	PIWA**K**IPHTD (615-624)	EIQ**K**ERS**K**RW (684-693)
AAV5	NGLD**R**GEPVN (56-65)	NPYL**K**YNHAD (87-96)	GMTNNLQGSN (510-519)	YLEGNMLITS (542-551)	ESETQPVNRV (552-561)	PIWA**K**IPETG (606-615)	ELK**K**ENS**K**RW (674-683)
AAV6	NGLD**K**GEPVN (57-66)	NPYL**R**YNHAD (88-97)	MASH**K**DDKDK (524-533)	TALDNVMITD (553-562)	EEEI**K**ATNPV (563-572)	PIWA**K**IPHTD (617-626)	ELQ**K**ENS**K**RW (686-695)
AAV7	NGLD**K**GEPVN (57-66)	NPYL**R**YNHAD (88-97)	MATH**K**DDEDR (526-535)	TTLENVLMTN (554-563)	EEEI**R**PTNPV (564-573)	PIWA**K**IPHTD (618-627)	ELQ**K**ENS**K**RW (687-696)
AAV8	NGLD**K**GEPVN (57-66)	NPYL**R**YNHAD A (88-97)	MATH**K**DDEER (526-535)	ADYSDVMLTS (553-562)	EEEI**K**TTNPV (565-574)	PIWA**K**IPHTD (619-628)	ELQ**K**ENS**K**RW (688-697)
AAV9	NGLD**K**GEPVN (57-66)	NPYL**K**YNHAD (88-97)	MASH**K**EGEDR (524-533)	VDAD**K**VMITN (553-562)	EEEI**K**TTNPV (563-572)	PIWA**K**IPHTD (617-626)	ELQ**K**ENS**K**RW (686-695)
AAV10	NGLD**K**GEPVN (57-66)	NPYL**R**YNHAD (88-197)	MATH**K**DDEER (526-535)	VDYSSVMLTS (555-564)	EEEI**K**TTNPV (565-574)	PIWA**K**IPHTD (619-628)	ELQ**K**ENS**K**RW (688-697)

Supplementary Table 3. Characterization of the number of labels per AAV

Amine-directed reaction	Vg (pmol)	NHS-Tz (nmol)	$(NOTA)_8$-A555-TCO (pmol)	n	ea (label)/Vg (particle) (mean±s.d.)
PHP.eB	1.2E12 (2)	0.4-0.7	20-40	6	5.4 ± 2.3
AAV9	2.0E12 (3.3)	0.7-1	30-50	8	3.5 ± 3.0
Thiol-directed reaction	Vg (pmol)	TCEP (nmol)	A555-C2-MI (pmol)	n	
AAV9-TC	1.7E12 (3)	0.3	60	4	0.5 ± 0.3

Supplementary Table 4. AAV clearance half-lives and 21-hour AUCs following tail vein administration in mice

Vector	Mouse strain	No. of mice	$t_{1/2}$ [a] (h)	R^2	$AUC_{0\text{-}21h}$ [b] (h.% ID/cc)
^{64}Cu-AAV9	C57BL/6	3	5.0	0.97	581.9
^{64}Cu-AAV9-TC	C57BL/6	3	2.4	0.99	344.3
^{64}Cu-PHP.eB	C57BL/6	3	3.1	0.96	391.1
	BALB/c	3	2.4	0.96	276.9
	BALB/c (neuraminidase)[c]	3	5.7	1.00	445.2
PHP.eB	C57BL/6	4	4.1	0.96	8.29[d]
$(NOTA)_8$-A555-PHP.eB	C57BL/6	4	5.3	0.94	8.88[d]

a. Adeno-associated virus clearance half-lives from blood, obtained from one-phase decay curve fit. b. Area under curve (AUC) from time radioactivity curve. c. Neuraminidase was administered to BALB/c mice through nose. d. Area under curve represents a fraction of remaining AAVs in blood

Supplementary Table 5. Radioactivity (% ID/cc) in blood pool measured from PET/CT images at 0, 4, and 21 h.

Time (h)	C57BL/6						BALB/c			
	^{64}Cu-AAV9 (n=3)		^{64}Cu-AAV9-TC (n=3)		^{64}Cu-PHP.eB (n=3)		^{64}Cu-PHP.eB (n=3)		^{64}Cu-PHP.eB (n=3, neuraminidase)	
	mean	s.d.	mean	s.d.	mean	s.d.	mean	s.d.	mean	s.d.
0	48.4	3.7	51.0	2.5	48.9	4.9	42.8	5.8	46.9	0.3
4	33.6	3.1	19.3	2.3	23.3	5.9	15.6	4.0	29.4	0.9
21	15.5	2.0	4.6	1.0	5.7	1.0	42.8	5.8	5.0	0.5

Supplementary Table 6. Radioactivity (% ID/cc) in whole brain measured from ROI analyses of PET/CT images at 0, 4, and 21 h.

Time (h)	C57BL/6						BALB/c			
	^{64}Cu-AAV9 (n=3)		^{64}Cu-AAV9-TC (n=3)		^{64}Cu-PHP.eB (n=3)		^{64}Cu-PHP.eB (n=3)		^{64}Cu-PHP.eB (n=3, neuraminidase)	
	mean	s.d.	mean	s. d.	mean	s. d.	mean	s. d.	mean	s. d.
0	3.94	0.39	4.21	0.27	10.7	3.1	2.81	0.53	3.46	0.38
4	3.03	0.70	2.31	0.06	19.2	4.0	1.30	0.37	2.22	0.14
21	1.91	0.31	1.40	0.12	15.8	3.9	0.78	0.06	0.98	0.10

Supplementary Table 7. Blood clearance of PHP.eB and $(NOTA)_8$-A555-PHP.eB assessed by classical PK analysis with qPCR.

Time (h)	Classical PK analysis					
	PHP.eB			$(NOTA)_8$-A555-PHP.eB		
	mean	s.d.	n	mean	s.d.	n
0	1	0	4	1	0	4
4	0.57	0.19	3	0.55	0.15	4
21	0.034	0.025	4	0.12	0.064	4

Mean values are presented as a fraction of the initial viral vector genome

Supplementary Table 8. Biodistribution of PHP.eB and $(NOTA)_8$-A555-PHP.eB with qPCR.

organs	PHP.eB			$(NOTA)_8$-A555-PHP.eB		
	mean	s.d.	n	mean	s.d.	n.
Brain	341.83	287.37	6	405.15	302.70	8
Heart	7.27	2.69	6	13.40	6.94	8
Liver	18.14	13.07	6	42.86	34.12	8
Spleen	208.33	311.67	6	137.06	114.43	8
Kidney	24.43	19.55	6	40.24	49.52	8
Blood	29443.33	17206.82	3	26225.00	4714.78	4

Mean values are presented as a relative viral gene number (Vg/Gc) with the viral vector gene concentration (Vg/mL) normalized by the glucagon housekeeping gene concentration (Gc/mL).

Supplementary Table 9. Biodistribution of AAVs in organs (% ID/cc) at 21 h and brain/blood ratio

	C57BL/6						BALB/c			
	^{64}Cu-AAV9 (n=3)		^{64}Cu-AAV9-TC (n=3)		^{64}Cu-PHP.eB (n=3)		^{64}Cu-PHP.eB (n=3)		^{64}Cu-PHP.eB (n=3, neuraminidase)	
	mean	s.d.	mean	s.d.	mean	s.d.	mean	s.d.	mean	s.d.
blood	16.9	3.6	3.6	0.3	2.1	0.3	1.7	0.2	2.0	0.2
heart	3.4	0.5	4.7	0.7	3.8	0.9	4.2	0.3	3.9	0.4
lungs	7.1	0.9	7.8	1.7	6.5	0.9	4.1	0.6	5.7	0.5
liver	18.3	1.2	19.3	1.8	35.1	10.4	37.7	1.7	44.4	0.8
spleen	15.8	3.3	6.0	0.5	39.4	9.6	18.3	1.8	18.8	1.4
kidneys	5.6	0.8	8.5	1.0	6.0	1.2	5.3	0.7	7.2	0.7
stomach	4.2	0.2	4.1	0.9	3.5	2.4	1.9	0.9	2.4	0.4
intestine	7.8	0.9	8.0	0.4	7.4	1.5	4.8	0.8	7.1	0.3
muscle	0.8	0.1	0.8	0.1	0.7	0.5	0.5	0.2	0.5	0.0
bone	2.8	0.6	2.2	0.3	2.4	0.7	1.3	0.3	1.6	0.2
brain	0.2	0.0	0.6	0.0	17.0	4.1	0.2	0.1	0.4	0.0
brain/blood	0.010	0.003	0.172	0.020	8.188	2.442	0.102	0.038	0.204	0.013

Supplementary Table 10. Radioactivity (% ID/cc) in liver measured from PET/CT images at 0, 4, and 21 h.

Time (h)	^{64}Cu-PHP.eB			
	C57BL/6 (n=3)		BALB/c (n=3)	
	mean	s.d.	mean	s.d.
0	26.9	5.3	33.3	4.5
4	30.5	7.4	45.0	2.7
21	25.7	6.6	37.0	3.2

Supplementary Table 11. AAVs and reporter genes

Experiment	Vector	Gene	Mouse strain	No. of mice	Data	preparation
In vitro	AAV9	*CAG-mNeonGreen*	N.A	N.A.	Fig. 2A-C	Caltech
	AAV9-TC	*CAG-mNeonGreen*	N.A	N.A	Fig. 2A-C, Supplementary Fig. 5	Caltech
	PHP.eB	*CAG-GFP*	N.A	N.A	Fig. 2A-C Supplementary Fig. 4	Addgene
In vivo transduction	PHP.eB	*CAG-GFP*	C57BL/6	6	Fig. 2D,E	Addgene
PET/CT	AAV9	*CAG-mNeonGreen*	C57BL/6	3	Fig. 3B-H	Caltech
	AAV9-TC	*CAG-mNeonGreen*	C57BL/6	3	Fig. 3B-H	Caltech
	PHP.eB	*CAG-GFP*	C57BL/6	3	Fig. 3B-H, Fig. 4A-F	Addgene
	PHP.eB	*CAG-mNeonGreen*	BALB/c	3	Fig. 4A-G	Caltech
	PHP.eB	*CAG-GFP*	BALB/c (neuraminidase)	4	Fig. 4G	Addgene
Optical	PHP.eB	*CAG-GFP*	C57BL/6	6	Supplementary Fig. 8, Movie 4	Addgene
	PHP.eB	*CAG-DIO-GFP*	C57BL/6	8	Fig. 3I	Caltech
	AAV9	*CAG-mNeonGreen*	C57BL/6		Supplementary Fig. 9, 10	Caltech
Classical PK and biodistribution	PHP.eB	*CAG-GFP*	C57BL/6	16	Fig. 3F	Addgene

Supplementary Figures

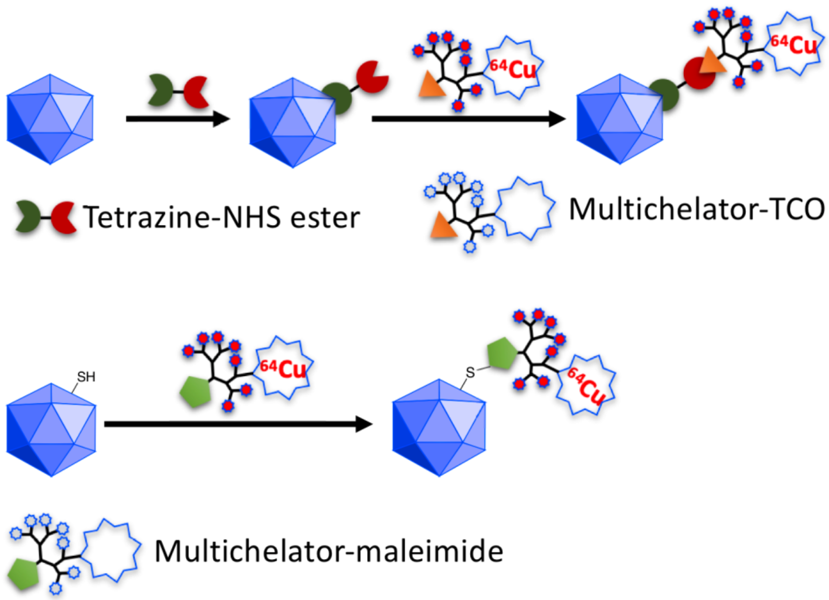

Supplementary Fig. 1. Schematic illustration of AAV radiolabeling via inverse electron demand Diels–Alder reaction (IEDDA) and maleimide-thiol reaction. Cu-64 is incorporated into a multichelator-TCO or multichelator-maleimide, following reaction with tetrazine-conjugated AAVs or with thiol, respectively. Both reactions afford a ^{64}Cu-labeled AAV with similar PK when tested with AAV9.

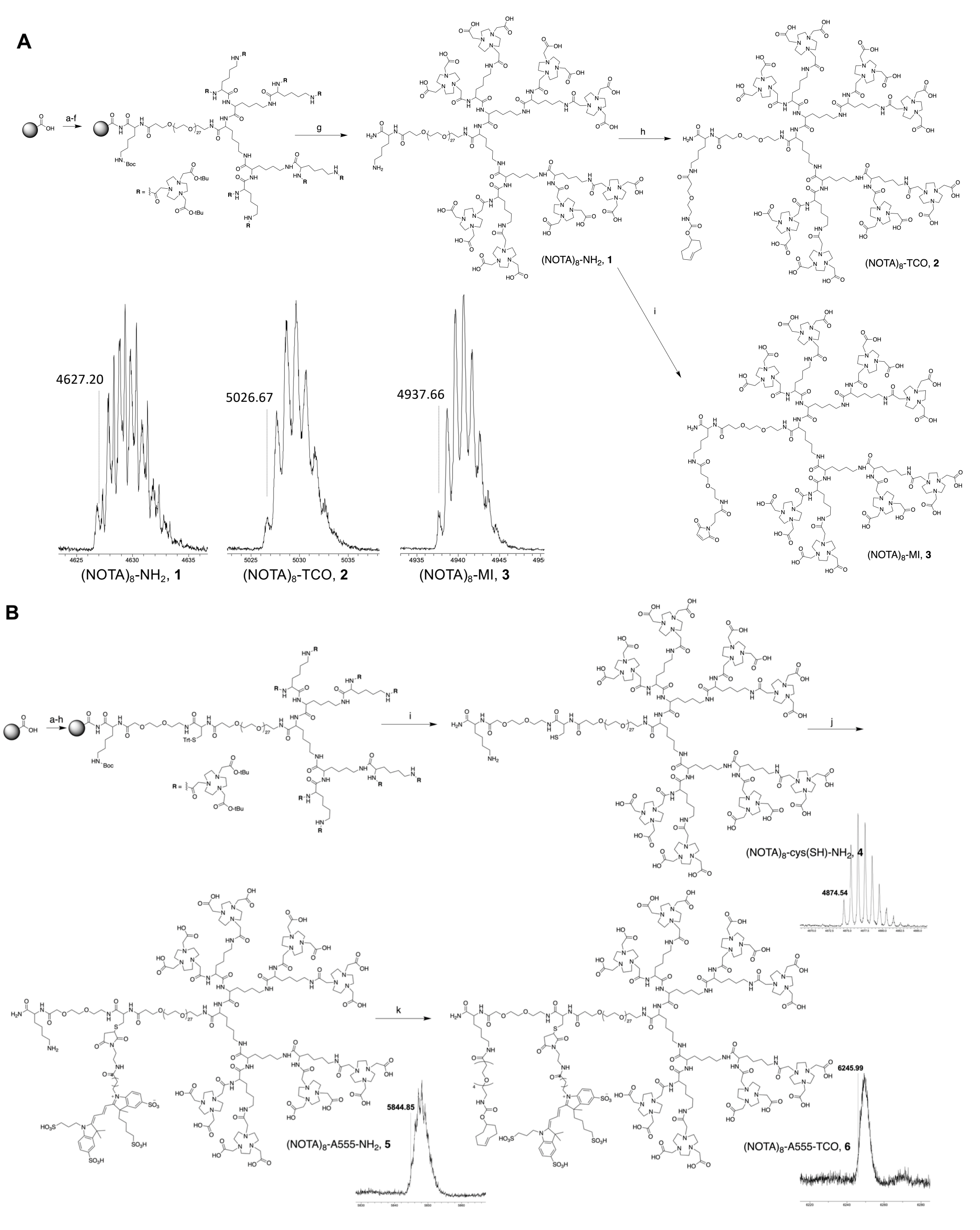

Supplementary Fig. 2. Scheme for multichelator synthesis and MALDI mass peaks of corresponding key products (1-6). (**A**) (a)Fmoc-Lys(Boc)-OH, (b) Fmoc-PEG$_{27}$-OH, (c-e) Fmoc-Lys(Fmoc)-OH, (f) *t*Bu-NOTA-OH, (g) TIPS (2.5%), H_2O (2.5%), TFA, (h) TCO-PEG$_4$-NHS/PBS, (i) NHS-PEG$_2$-MI/PBS; reagents for a-f coupling reaction: HBTU, DIPEA in DMF, for Fmoc removal: 20% 4-methylpiperidine in DMF. (**B**) (a) Fmoc-Lys(Boc)-OH, (b) Fmoc-PEG-OH, (c) Fmoc-Cys(Trt)-OH, (d) Fmoc-PEG$_{27}$-OH, (e-g) Fmoc-Lys(Fmoc)-OH, (h) tBu-NOTA-OH, (i) TIPS (2.5%), H_2O (2.5%), TFA, (j) AF555-maleimide/PBS (pH 7.0-7.5), (k) TCO-PEG$_4$-NHS/PBS; reagents for a-h coupling reaction: HBTU, DIPEA in DMF, for Fmoc removal: 20% 4-methylpiperidine in DMF. AF555 is denoted as A555 at Fig. 3F, 3I and Supplementary Fig. 7A.

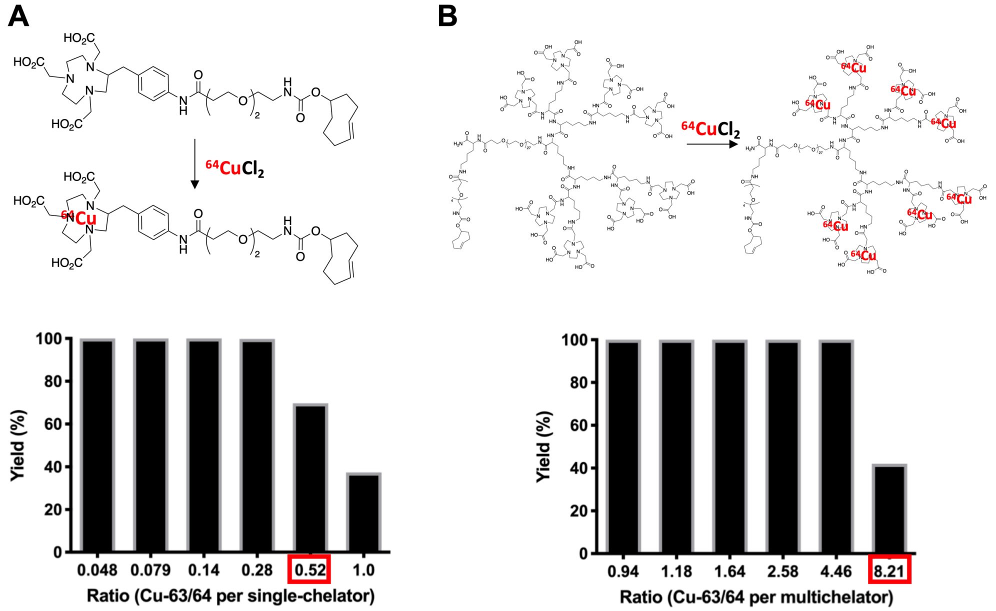

Supplementary Fig. 3. The multichelator incorporates ~10 times higher molar radioactivity than the single chelator. Cu-63/64 titration with the (**A**) single-chelator (NOTA-TCO) and (**B**) multichelator $(NOTA)_8$-TCO. Titration results show that one multichelator was able to chelate five to eight Cu-63/64 whereas two NOTA-TCOs were required to incorporate one Cu-63/64.

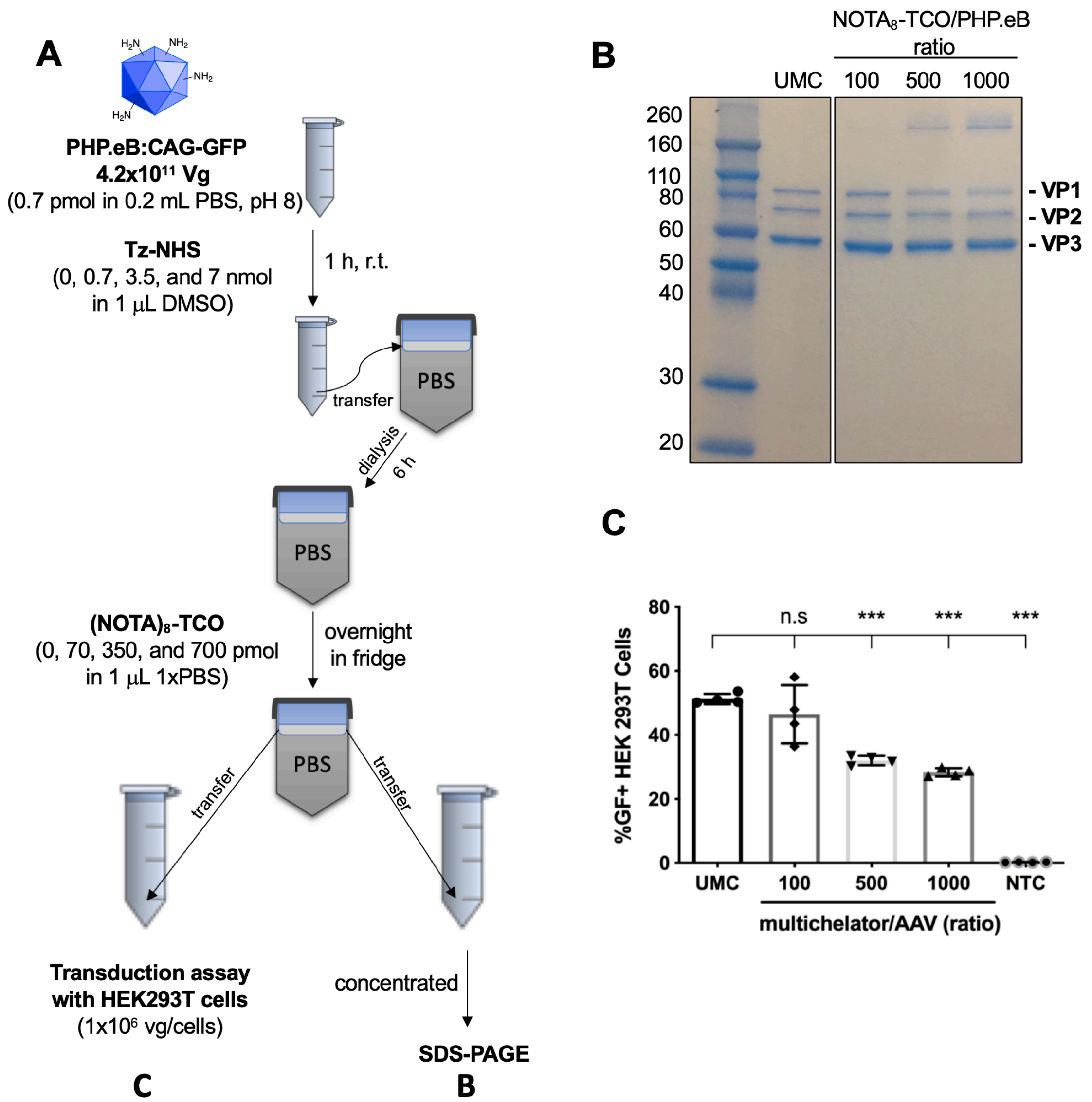

Supplementary Fig. 4. Optimization of PHP.eB:*CAG-GFP* labeling with $(NOTA)_8$-TCO. (**A**) Experimental scheme for the optimization of PHP.eB labeling via IEDDA reaction. (**B**) SDS-PAGE of PHP.eB capsid after surface lysine modification with Tz-NHS ester and labeling with $(NOTA)_8$-TCO at 100, 500 and 1000 mole ratio. Gel image is from a single gel and experiment. (**C**) Percentage of green fluorescent positive (GF+) HEK293T cells at 2 days after incubation with the surface modified PHP.eB:*CAG-GFP* at 100, 500 and 1000 mole ratio of $(NOTA)_8$-TCO (n=4 per group). The frequency of GF^+ cells in all cell samples incubated with unmodified control (UMC) PHP.eB was compared with cells incubated with modified PHP.eBs at 100 (black diamond, n.s), 500 (black reverse triangle, $P<0.0001$), and 1000 (black triangle, $P<0.0001$) mole ratio of $(NOTA)_8$-TCO. Data are shown as mean ± SD. Ordinary one-way ANOVA with Dunnett's multiple comparison test were used to compare UMC with modified PHP.eB groups and non-treated control (NTC). Statistical significance is presented as n.s. (not significant) and *** $P \le 0.001$.

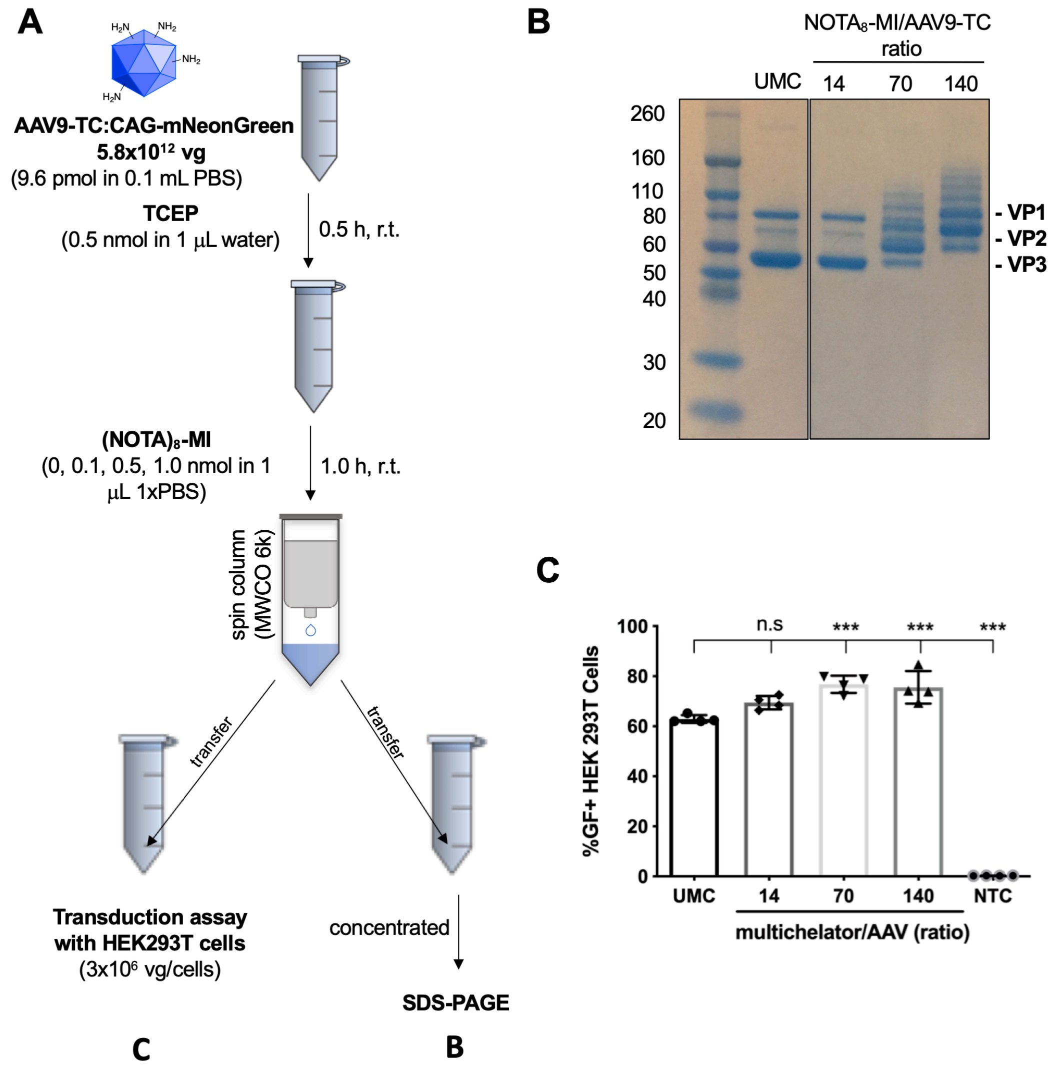

Supplementary Fig. 5. Optimization of AAV9-TC:*CAG-mNeonGreen* labeling with $(NOTA)_8$-MI. (**A**) Experimental scheme for the optimization of the AAV9-TC labeling reaction. (**B**) SDS-PAGE of AAV9-TC capsid after conjugation with $(NOTA)_8$-MI at 14, 70 and 140 mole ratio. Gel image from a single gel and experiment. (**C**) Percentage of green fluorescent positive (GF^+) HEK293T cells 2 days after incubation of AAV9-TC:*CAG-mNeonGreen* at 14, 70 and 140 mole ratio of $(NOTA)_8$-MI (n=4 per group). The frequency of GF^+ cells in all cell samples incubated with unmodified control (UMC) AAV9-TC was compared with cells incubated with modified AAV9-TCs at 14 (black diamond, n.s), 70 (black reverse triangle, P=0.0002), and 140 (black triangle, P=0.0005) mole ratio of $(NOTA)_8$-MI. Data are shown as mean ± SD. Ordinary one-way ANOVA with Dunnett's multiple comparison test was used to compare UMC with modified AAV9-TC groups and non-treated control (NTC). Statistical significance is presented as n.s. (not significant) and *** $P \leq 0.001$.

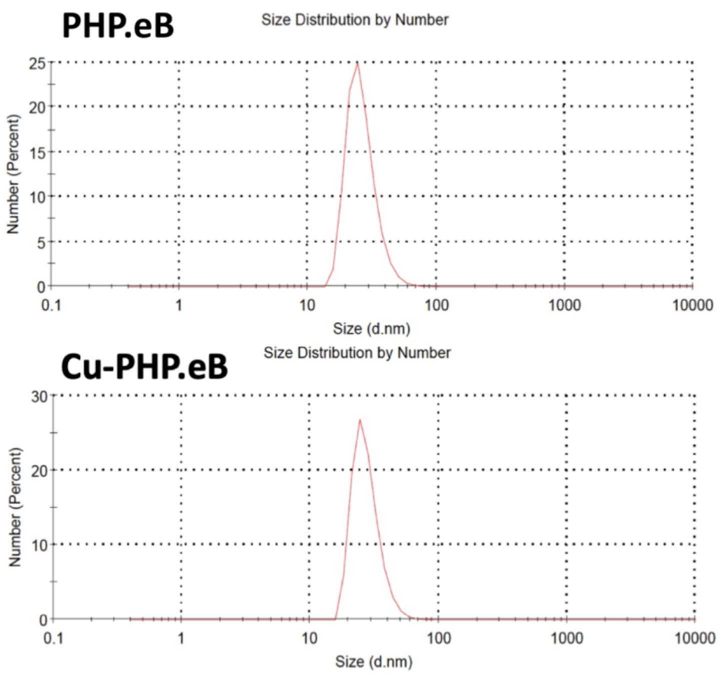

Supplementary Fig. 6. The size distribution of Cu-labeled and unlabeled PHP.eB.

A

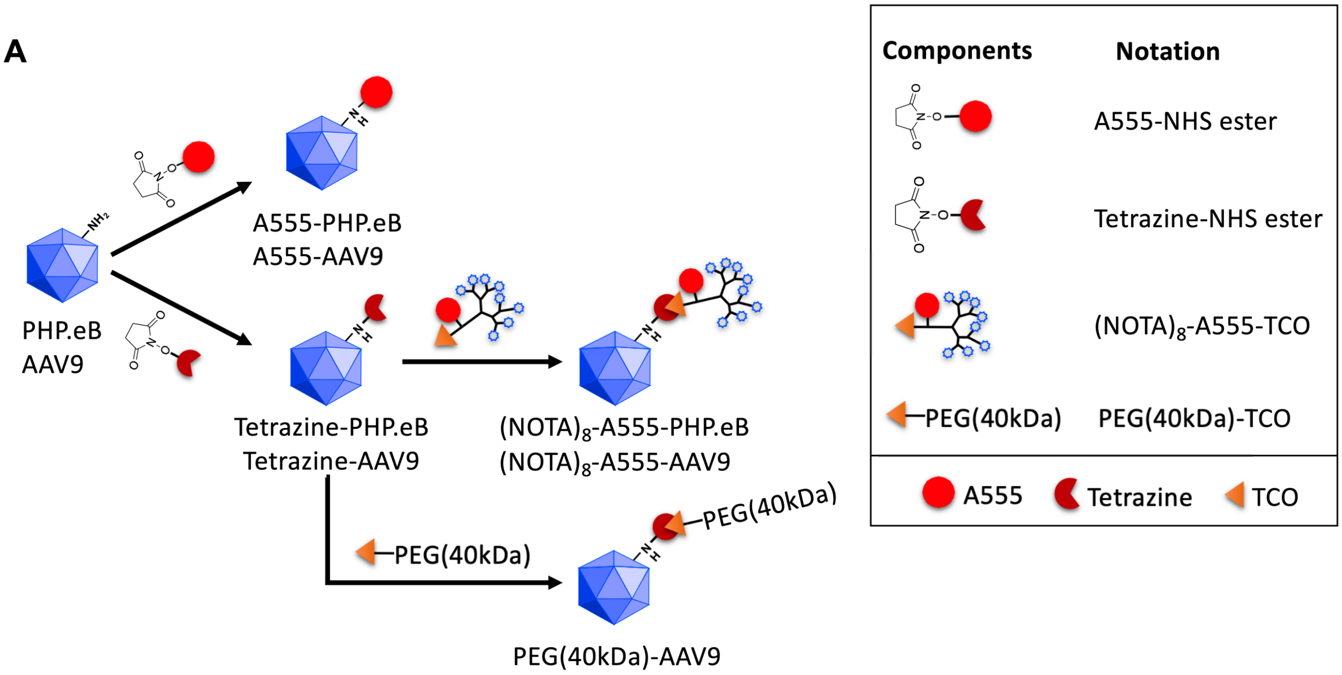

B

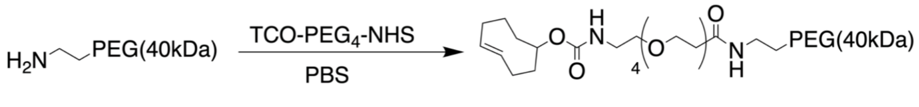

Supplementary Fig. 7. Schematic view of optical labeling of PHP.eB and 40kDa PEG conjugation. (**A**) Schematic illustration of optical labeling of PHP.eB and AAV9. A555-NHS ester was coupled to surface amines on PHP.eB or AAV9 (upper path), and (NOTA)$_8$-A555-TCO was conjugated to PHP.eB or AAV9 to form (NOTA)$_8$-A555-PHP.eB or (NOTA)$_8$-A555-AAV9 (middle path) as an analog of the multichelator used in the PET studies. PEG(40kDa)-TCO was conjugated to AAV9 to form PEG(40kDa)-AAV9 (bottom path). (**B**) Scheme for the synthesis of PEG(40kDa)-TCO.

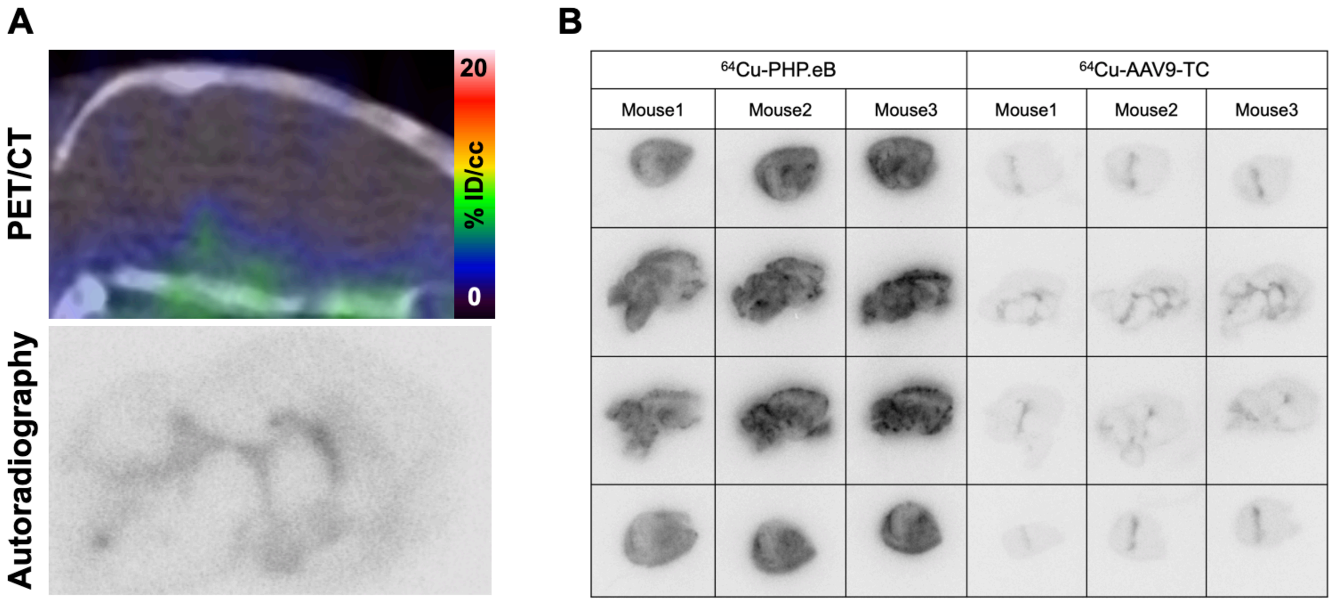

Supplementary Fig. 8. PET/CT and autoradiography images from the brain of mice injected with radiolabeled AAVs. (**A**) Sliced PET/CT and autoradiography of sagittal section of mouse brain acquired at 21 h after tail vein injection of ^{64}Cu-AAV9-TC. Images represent a control to be compared with ^{64}Cu-PHP.eB in Fig. 3. (**B**) Autoradiography images of sagittally-sliced mouse brains acquired at 21 h after tail vein injection of ^{64}Cu-PHP.eB (three left columns) and ^{64}Cu-AAV9-TC (three right columns). The autoradiography results in Supplementary Fig. 8 result from a single experiment while PET imaging of ^{64}Cu-PHP.eB was repeated in two experiments and ^{64}Cu-AAV9-TC in one experiment.

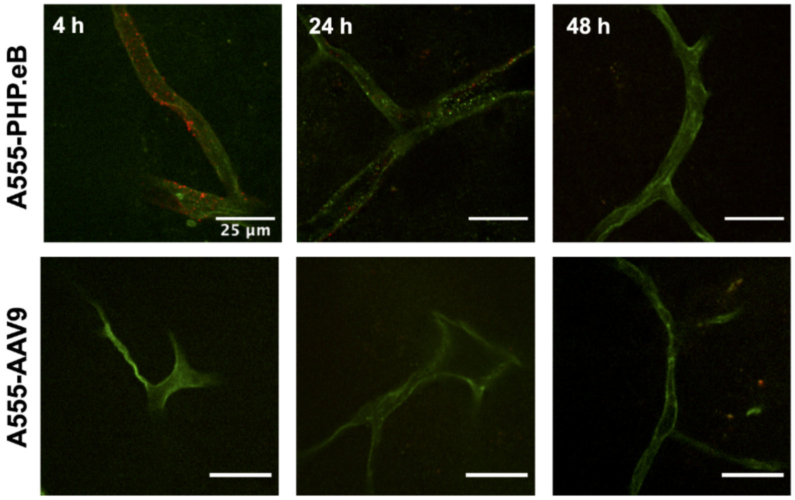

Supplementary Fig. 9. Brain endothelial images acquired at 4, 24 and 48 h after tail vein administration of A555-PHP.eB or A555-AAV9. Green and red fluorescence represent brain endothelium and PHP.eB, respectively. Endothelium was stained with FITC-lectin. Representative images were chosen from mice (A555-PHP.eB) euthanized at 4 (n=3), 24 (n=2) and 48 h (n=1) and mice (A555-AAV9) at 4 (n=2), 24 (n=1) and 48 h (n=1). Representative fluorescence images with A555-PHP.eB were chosen from mice euthanized at 4 (two experiments), 24 (one experiment) and 48 h (one experiment) and for A555-AAV9 in one experiment.

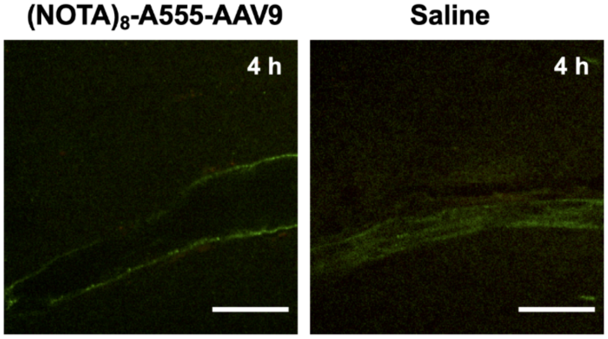

Supplementary Fig. 10. Optical images of brain endothelium acquired at 4 h after tail vein administration of $(NOTA)_8$-A555-AAV9 or saline. Green and red fluorescence represent brain endothelium and AAVs respectively. The results can be compared with the injection of $(NOTA)_8$-A555-PHP.eB shown in Fig. 3. Representative images were chosen from mice ($(NOTA)_8$-A555-AAV9 and saline) euthanized at 4 h (n=2 and n=2, respectively) from a single experiment.

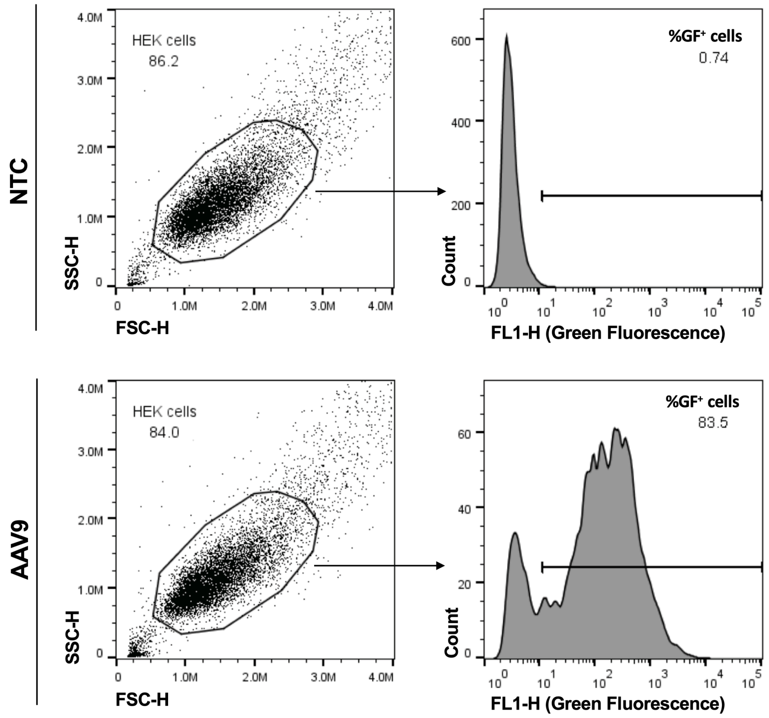

Supplementary Fig. 11. Optical gating strategy used to determine the percentage of green fluorescent HEK293 cells presented in Fig. 2B and Supplementary Fig. 4C, 5C. Typical non-treated (NT) and AAV9-treated (AAV9) cell samples are presented.

Supplementary References

1. Challis, R. C. *et al.* Systemic AAV vectors for widespread and targeted gene delivery in rodents. *Nat. Protoc.* **14**, 379–414 (2019).
2. Chandran, J. S. *et al.* Site Specific Modification of Adeno-Associated Virus Enables Both Fluorescent Imaging of Viral Particles and Characterization of the Capsid Interactome. *Sci Rep* **7**, 14766 (2017).
3. Bartlett, J. S. & Samulski, R. J. Fluorescent viral vectors: A new technique for the pharmacological analysis of gene therapy. *Nat. Med.* **4**, 635–637 (1998).
4. Bartlett, J. S., Wilcher, R. & Samulski, R. J. Infectious entry pathway of adeno-associated virus and adeno-associated virus vectors. *J. Virol.* **74**, 2777–2785 (2000).
5. Liu, Y., Joo, K. I. & Wang, P. Endocytic processing of adeno-associated virus type 8 vectors for transduction of target cells. *Gene Ther.* **20**, 308–317 (2013).
6. Sanlioglu, S. *et al.* Endocytosis and nuclear trafficking of adeno-associated virus type 2 are controlled by Rac1 and phosphatidylinositol-3 kinase activation. *J. Virol.* **74**, 9184–9196 (2000).
7. Xiao, W., Warrington, K. H., Hearing, P., Hughes, J. & Muzyczka, N. Adenovirus-facilitated nuclear translocation of adeno-associated virus type 2. *J. Virol.* **76**, 11505–11517 (2002).
8. Lee, G. K., Maheshri, N., Kaspar, B. & Schaffer, D. V. PEG conjugation moderately protects adeno-associated viral vectors against antibody neutralization. *Biotechnol. Bioeng.* **92**, 24–34 (2005).
