# Supplementary material for: Positron emission tomography imaging of novel AAV capsids maps rapid brain accumulation
Source: Nat Commun. 2020 Apr 30;11:2102. doi: 10.1038/s41467-020-15818-4 (PMC7193641; doi:10.1038/s41467-020-15818-4)
Supplement: Supplementary file 2 — Description of Additional Supplementary Files [file 41467_2020_15818_MOESM2_ESM.pdf]

Description of Additional Supplementary Files

File Name: Supplementary Movie 1
Description: Projected PET/CT images acquired at (a) 4 and (b) 21 hours from mouse injected with ^{64}Cu-AAV9.

File Name: Supplementary Movie 2
Description: Projected PET/CT images acquired at (a) 4 and (b) 21 hours from mouse injected with ^{64}Cu-PHP.eB.

File Name: Supplementary Movie 3
Description: Projected PET/CT images acquired at (a) 4 and (b) 21 hours from mouse injected with ^{64}Cu-AAV9-TC.

File Name: Supplementary Movie 4
Description: Z-stack movie clip of brain endothelium images acquired at 4 hours from mice injected (a) A555- and (b) $(NOTA)_8$-A555-PHP.eB.
