# Supplementary material for: Positron emission tomography imaging of novel AAV capsids maps rapid brain accumulation
Source: Nat Commun. 2020 Apr 30;11:2102. doi: 10.1038/s41467-020-15818-4 (PMC7193641; doi:10.1038/s41467-020-15818-4)
Supplement: Supplementary file 11 — Reporting Summary [file 41467_2020_15818_MOESM11_ESM.pdf]

nature research

Corresponding author(s): Katherine Ferrara

Last updated by author(s): March 21, 2020

Reporting Summary

Nature Research wishes to improve the reproducibility of the work that we publish. This form provides structure for consistency and transparency in reporting. For further information on Nature Research policies, see Authors & Referees and the Editorial Policy Checklist.

Statistics

For all statistical analyses, confirm that the following items are present in the figure legend, table legend, main text, or Methods section.

n/a	Confirmed	
☐	☒	The exact sample size (n) for each experimental group/condition, given as a discrete number and unit of measurement
☐	☒	A statement on whether measurements were taken from distinct samples or whether the same sample was measured repeatedly
☐	☒	The statistical test(s) used AND whether they are one- or two-sided<br>*Only common tests should be described solely by name; describe more complex techniques in the Methods section.*
☐	☒	A description of all covariates tested
☐	☒	A description of any assumptions or corrections, such as tests of normality and adjustment for multiple comparisons
☐	☒	A full description of the statistical parameters including central tendency (e.g. means) or other basic estimates (e.g. regression coefficient) AND variation (e.g. standard deviation) or associated estimates of uncertainty (e.g. confidence intervals)
☐	☒	For null hypothesis testing, the test statistic (e.g. F, t, r) with confidence intervals, effect sizes, degrees of freedom and P value noted<br>*Give P values as exact values whenever suitable.*
☒	☐	For Bayesian analysis, information on the choice of priors and Markov chain Monte Carlo settings
☒	☐	For hierarchical and complex designs, identification of the appropriate level for tests and full reporting of outcomes
☒	☐	Estimates of effect sizes (e.g. Cohen's d, Pearson's r), indicating how they were calculated

Our web collection on statistics for biologists contains articles on many of the points above.

Software and code

Policy information about availability of computer code

Data collection	PET/CT data was collected with Inveon Acquisition Workspace (Siemens) . Microscope data collected with LAS X (Leica) and SlideBook6 (3i). qPCR data collected from CFX Maestro (Bio-Rad)
Data analysis	Inveon Reseach Workplace 4.2 (IRW, Siemens) was used for PET/CT data analysis. Microsoft Excel (ver. 16.35), and GraphPad Prism 8 are used for general data and statistical analysis. ImageJ (ver. 2.0). PyMOL 2.0 (Molecular Graphics System) was used to process the structure of capsid. FlowJo v10.1 (Treestar) was used for data analyses of results from flow cytometry.

For manuscripts utilizing custom algorithms or software that are central to the research but not yet described in published literature, software must be made available to editors/reviewers. We strongly encourage code deposition in a community repository (e.g. GitHub). See the Nature Research guidelines for submitting code & software for further information.

Data

Policy information about availability of data

All manuscripts must include a data availability statement. This statement should provide the following information, where applicable:

- Accession codes, unique identifiers, or web links for publicly available datasets
- A list of figures that have associated raw data
- A description of any restrictions on data availability

The Mus musculus database from the Universal Protein Resource (UniProt, http://www.uniprot.org) along with the sequences of capsid proteins was used for the process of the raw data fiiles from mass spectrometer. Protein Data Bank (PDB ID:3Ux1) was used to display capsid structure. The authors declare that image and quantitative data supporting the findings of this study are available within the paper and its supplementary information files. The raw PET images and associated data that support the findings of this study are available from the corresponding author upon reasonable request.

Field-specific reporting

Please select the one below that is the best fit for your research. If you are not sure, read the appropriate sections before making your selection.

☒ Life sciences ☐ Behavioural & social sciences ☐ Ecological, evolutionary & environmental sciences

For a reference copy of the document with all sections, see nature.com/documents/nr-reporting-summary-flat.pdf

Life sciences study design

All studies must disclose on these points even when the disclosure is negative.

Sample size	Sample size of each group in experiment is presented in results and methods.
Data exclusions	One data point at 4 hours in Fig 3F was excluded due to the insufficient volume of blood collected. One tail vein injection failed in a mouse injected with PHP.eB. This animal was excluded from data in Fig 3F left bar graph.
Replication	Replication is described in the Statistics and Reproducibility section.
Randomization	Allocation was random.
Blinding	The technicians who performed the studies were blinded to the biological groups.

Reporting for specific materials, systems and methods

We require information from authors about some types of materials, experimental systems and methods used in many studies. Here, indicate whether each material, system or method listed is relevant to your study. If you are not sure if a list item applies to your research, read the appropriate section before selecting a response.

Materials & experimental systems

n/a	Involved in the study	
☒	☐	Antibodies
☐	☒	Eukaryotic cell lines
☒	☐	Palaeontology
☐	☒	Animals and other organisms
☒	☐	Human research participants
☒	☐	Clinical data

Methods

n/a	Involved in the study	
☒	☐	ChIP-seq
☐	☒	Flow cytometry
☒	☐	MRI-based neuroimaging

Eukaryotic cell lines

Policy information about cell lines

Cell line source(s)	ATCC (HEK 293T)
Authentication	Karyotyping, morphology, and STR testing for authentication on the ventor's end (ATTC). Information on karyotyping and morphology is located here: https://www.atcc.org/products/all/CRL-1573.aspx#characteristics. In addition, ATCC does short tandem repeat (STR) profiling to confirm cell identity - which for our cells is located here: https://www.atcc.org/products/all/CRL-1573.aspx#specifications
Mycoplasma contamination	PCR of media incubated with these cells returned a negative result for mycoplasma contamination
Commonly misidentified lines (See ICLAC register)	No commonly misidentified cell lines were used in the study.

Animals and other organisms

Policy information about studies involving animals; ARRIVE guidelines recommended for reporting animal research

Laboratory animals	C57BL/6, BALB/c female mice were purchased from Jackson Laboratory. Average age of mice was 66 days (+-25 days). Mice were kept at room temperature 20-24 'C, humidity 40-60% and 12 hour light/12 dark cycle.
Wild animals	No wild animals were used in the study.

Field-collected samples	No field collected samples were used in the study.
Ethics oversight	All animal experiments were conducted under an animal use protocol approved by the University of California, Davis, Institutional Animal Care and Use Committee (IACUC) and Stanford University, Administrative Panel on Laboratory Animal Care (APLAC)

Note that full information on the approval of the study protocol must also be provided in the manuscript.

Flow Cytometry

Plots

Confirm that:

☒ The axis labels state the marker and fluorochrome used (e.g. CD4-FITC).

☒ The axis scales are clearly visible. Include numbers along axes only for bottom left plot of group (a 'group' is an analysis of identical markers).

☒ All plots are contour plots with outliers or pseudocolor plots.

☒ A numerical value for number of cells or percentage (with statistics) is provided.

Methodology

Sample preparation	Sample Preparation procedure is described in Methods.
Instrument	Flow cytometer (BD FACScan)
Software	FlowJo (BD)
Cell population abundance	We did not sort cells with these experiments. We collected and assayed the entire population of cultured HEK cells.
Gating strategy	The preliminary FSC/SSC bivariate plot was created in order to eliminate debris (events below 10^3 on the FSC-H log scale and 10^2 on the SSC-H log scale) and specifically select only the HEK 293 cultured cell population using an elliptical gate. After selecting only the HEK293 cell population, a single-parameter histogram plot was generated to analyze GFP fluorescence intensity signal in each cell (FL1-H detection channel) along the X-axis and event count along the Y-axis. A range gate was then placed that included any cells expressing signal in the FL1-H detection channel (GFP signal) above background (background fluorescence signal in the FL1-H channel was determined from the no-treatment control (NTC) samples). The gating figure was added.

☒ Tick this box to confirm that a figure exemplifying the gating strategy is provided in the Supplementary Information.
